# Prognostic factors to identify resolution of small bowel obstruction without need for operative management: systematic review

**DOI:** 10.1007/s00330-023-10421-9

**Published:** 2023-11-08

**Authors:** Vivienne N. Eze, Tom Parry, Darren Boone, Sue Mallett, Steve Halligan

**Affiliations:** Centre for Medical Imaging, University College London UCL, Charles Bell House, 43-45 Foley Street, London, W1W 7TS UK

**Keywords:** CT, Intestinal obstruction, Intestinal ischaemia, Systematic review, Meta-analysis

## Abstract

**Objectives:**

To identify imaging, clinical, and laboratory variables potentially prognostic for surgical management of small bowel obstruction.

**Methods:**

Two researchers systematically reviewed indexed literature 2001–2021 inclusive for imaging, clinical, and laboratory variables potentially predictive of surgical management of small bowl obstruction and/or ischaemia at surgery, where performed. Risk of bias was assessed. Contingency tables for variables reported in at least 5 studies were extracted and meta-analysed to identify strong evidence of association with clinical outcomes, across studies.

**Results:**

Thirty-one studies were ultimately included, reporting 4638 patients (44 to 313 per study). 11 (35%) studies raised no risk of bias concerns. CT was the modality reported most (29 studies, 94%). Meta-analysis of 21 predictors identified 5 strongly associated with surgical intervention, 3 derived from CT (peritoneal free fluid, odds ratio [OR] 3.24, 95%CI 2.45 to 4.29; high grade obstruction, OR 3.58, 95%CI 2.46 to 5.20; mesenteric inflammation, OR 2.61, 95%CI 1.94 to 3.50; abdominal distension, OR 2.43, 95%CI 1.34 to 4.42; peritonism, OR 3.97, 95%CI 2.67 to 5.90) and one with conservative management (previous abdominopelvic surgery, OR 0.58, 95%CI 0.40 to 0.85). Meta-analysis of 10 predictors identified 3 strongly associated with ischaemia at surgery, 2 derived from CT (peritoneal free fluid, OR 3.49, 95%CI 2.28 to 5.35; bowel thickening, OR 3.26 95%CI 1.91 to 5.55; white cell count, OR 4.76, 95%CI 2.71 to 8.36).

**Conclusions:**

Systematic review of patients with small bowel obstruction identified four imaging, three clinical, and one laboratory predictors associated strongly with surgical intervention and/or ischaemia at surgery.

**Clinical relevance statement:**

Via systematic review and meta-analysis, we identified imaging, clinical, and laboratory predictors strongly associated with surgical management of small bowel obstruction and/or ischaemia. Multivariable model development to guide management should incorporate these since they display strong evidence of potential utility.

**Key Points:**

• *While multivariable models incorporating clinical, laboratory, and imaging factors could predict surgical management of small bowel obstruction, none are used widely.*

• *Via systematic review and meta-analysis we identified imaging, clinical, and laboratory variables strongly associated with surgical management and/or ischaemia at surgery.*

• *Development of multivariable models to guide management should incorporate these predictors, notably CT scanning, since they display strong evidence of potential utility.*

**Supplementary Information:**

The online version contains supplementary material available at 10.1007/s00330-023-10421-9.

## Introduction

Small bowel obstruction (SBO) is common: It accounts for 20% of abdominal surgery in patients presenting with acute abdominal pain [1], and around one-quarter of SBO admissions culminate in surgery [2]. In the developed world, adhesions remain the most common cause. The limitations of plain abdominal radiography are now appreciated widely, and a seminal 1991 paper transitioned diagnosis towards CT scanning [3], which determines both the cause and level of obstruction more accurately. With improved diagnosis by CT, the pivotal clinical question then becomes whether surgery is necessary or not? The old surgical maxim of, “Never let the sun set on a small bowel obstruction,” has been replaced by a shift towards more conservative management, with around 75% of patients now avoiding an operation [2]. Nevertheless, untreated obstruction can culminate in irreversible mural ischaemia and intestinal perforation, a surgical catastrophe with considerable morbidity and mortality. The dilemma familiar to every general surgeon is thus: Operate too soon and expose the patient to unnecessary surgical risk; too late, and the patient is in extremis.

It is surprising that SBO prognostication still adopts a “try it and see” approach, bolstered by surgical experience. Failed conservative treatment, signs of peritonism, and clinical concerns for ischaemia may precipitate surgery [2]. While it is seemingly intuitive that a multivariable prognostic model incorporating clinical, laboratory, and imaging factors could predict surgical requirement, no models are used widely. While some authors have investigated radiological predictors of surgery, they have largely excluded clinical and laboratory predictors [4–11]. However, it is highly unlikely that surgeons will adopt a model that ignores fundamental clinical factors [12]. Accordingly, we performed a systematic review of clinical, laboratory, and imaging factors that might predict SBO resolution without the need for surgery. We then meta-analysed potential predictors to identify those most likely to contribute usefully to model development.

## Materials and methods

Our institution does not require ethical permission for secondary research using primary literature. The research is reported according to the Preferred Reporting terms for Systematic Reviews and Meta-analyses (PRISMA) [13].

### Target condition, search strategy, and study selection

We wrote a protocol and then developed and piloted a search string to identify imaging, clinical, and laboratory variables (including existing models) potentially predictive of SBO resolution without operative management (Online supplementary material 1). We used terms to identify studies of bowel obstruction/ileus. We included terms to identify prognostic research. We limited to adults and excluded narrative reviews, editorials, letters, etc. We searched the US National Library of Medicine PUBMED journal citation database (http://www.ncbi.nlm.nih.gov/pubmed) from 2001 to 2021 inclusive. This search was supplemented by a subsequent review of references cited by included studies. The search was performed in September 2021 by two radiologists (VE, DB), one of whom had > 10 years of experience in systematic review design, data extraction, and analysis.

### Inclusion/exclusion criteria

We included studies investigating imaging variables potentially predictive of SBO resolution without the need for operative management (or, depending on how the research was framed, factors that predicted surgical intervention). We excluded studies limited to colonic obstruction; solely paediatric studies; studies solely of malignant obstruction; studies limited to early post-operative obstruction (a separate entity with different aetiology); studies limited to inflammatory bowel disease or chronic functional obstruction. We did not specifically set out to exclude studies that did not include intravenous contrast for CT scanning because we anticipated that the large majority would administer contrast, and contrast only influences one potential predictor variable, mucosal enhancement. We also anticipated that any representative consecutive series would include some patients in whom IV contrast is contraindicated but from whom other potential predictors could be extracted. We excluded studies reporting less than five patients in either operative or conservative groups, since these are underpowered.

### Data screening and extraction

Potential studies were identified via scrutiny of the online title and abstract, and definite exclusions were discarded. The full text was obtained for those remaining potentially eligible. Uncertainty was resolved by face-to-face consensus meetings between all authors. Mindful of the distinction between prognostic and diagnostic data [12] and aware that studies frequently confound these, we piloted extraction on the initial 10 studies identified to ascertain potential literature quality and thenceforth review viability. We found data could be categorised into five broad headings: study design, patient characteristics, radiological predictors, non-radiological predictors (further subdivided into clinical or laboratory predictors), and clinical outcomes. Thereby informed, we developed an extraction sheet (Microsoft Excel) populated subsequently by selected studies as follows: Study design, patient characteristics (demographics), imaging variables, clinical variables, laboratory variables, and the overall outcome (surgery vs. conservative treatment, expressed as contingency tables). If intra-operative findings such as the presence of ischaemia/strangulation were reported, or if resection was performed, we also extracted these. We noted whether the authors had attempted to develop a predictive score or model.

### Risk of bias and applicability assessment

We assessed the study methodological quality and potential sources of bias using a modified Quality Assessment of Diagnostic Accuracy Studies (QUADAS 2) tool [14]. This consisted of the four main domains that assessed patient selection, index tests, reference standards, and patient flow through the study. QUADAS applies to diagnostic studies, so we adapted for prognosis via three additional questions: (1) Did sufficient participants exhibit the primary outcome of interest (defined as > 20 events per study); (2) Were at least three standard clinical variables reported in addition to imaging factors and, if not, did authors justify this; (3) Were at least three predictor estimates reported with non-statistically significant results? We assigned categories of “low/high/unclear.”

### Analysis

Extracted data were analysed and expressed as simple summary statistics. We intended to meta-analyse both imaging and non-imaging predictor variables where sufficient data were presented. We excluded predictors reported by less than five individual studies to avoid under-powered meta-analysis. We anticipated heterogenous data and sought predictor association with conservative or operative outcomes rather than precise estimates of strength or interpredictor comparisons. Also, because of anticipated heterogeneity, we intended meta-analysis to reflect general evidence across studies rather than providing precise estimates regarding specific definitions, situations, measurements, and thresholds. Data were extracted as 2 × 2 tables or univariable odds ratios (OR); 2 × 2 results were converted into ORs for meta-analysis. A random-effects meta-analysis used methods of DerSimonian and Laird, with the estimate of heterogeneity taken from the inverse-variance fixed-effect model. Summaries across predictors and individual study results for each predictor were presented as forest plots. High ORs may be due to a small sample size and/or study bias, so we investigated additional factors to determine the strength of evidence for each predictor rather than relying simply on statistical significance. First, two medical statisticians examined the width of the 95% confidence intervals (CI) around the overall effect of each predictor because narrow CIs indicate greater statistical power behind evidence. They then identified predictors where the OR point estimate was consistently above or below 1.0 across all the individual studies that were meta-analysed, indicating reliable results. They also considered whether the OR was “credible” because ORs far removed from 1 usually indicate low statistical power or unreliable evidence.

During extraction, it became apparent that some studies reported outcomes as ischaemia/no ischaemia rather than surgery/no surgery. We therefore analysed these outcomes separately. Two medical statisticians (TP, SM) used STATA 14.2 (StataCorp) for meta-analysis.

## Results

**Fig. 1:**
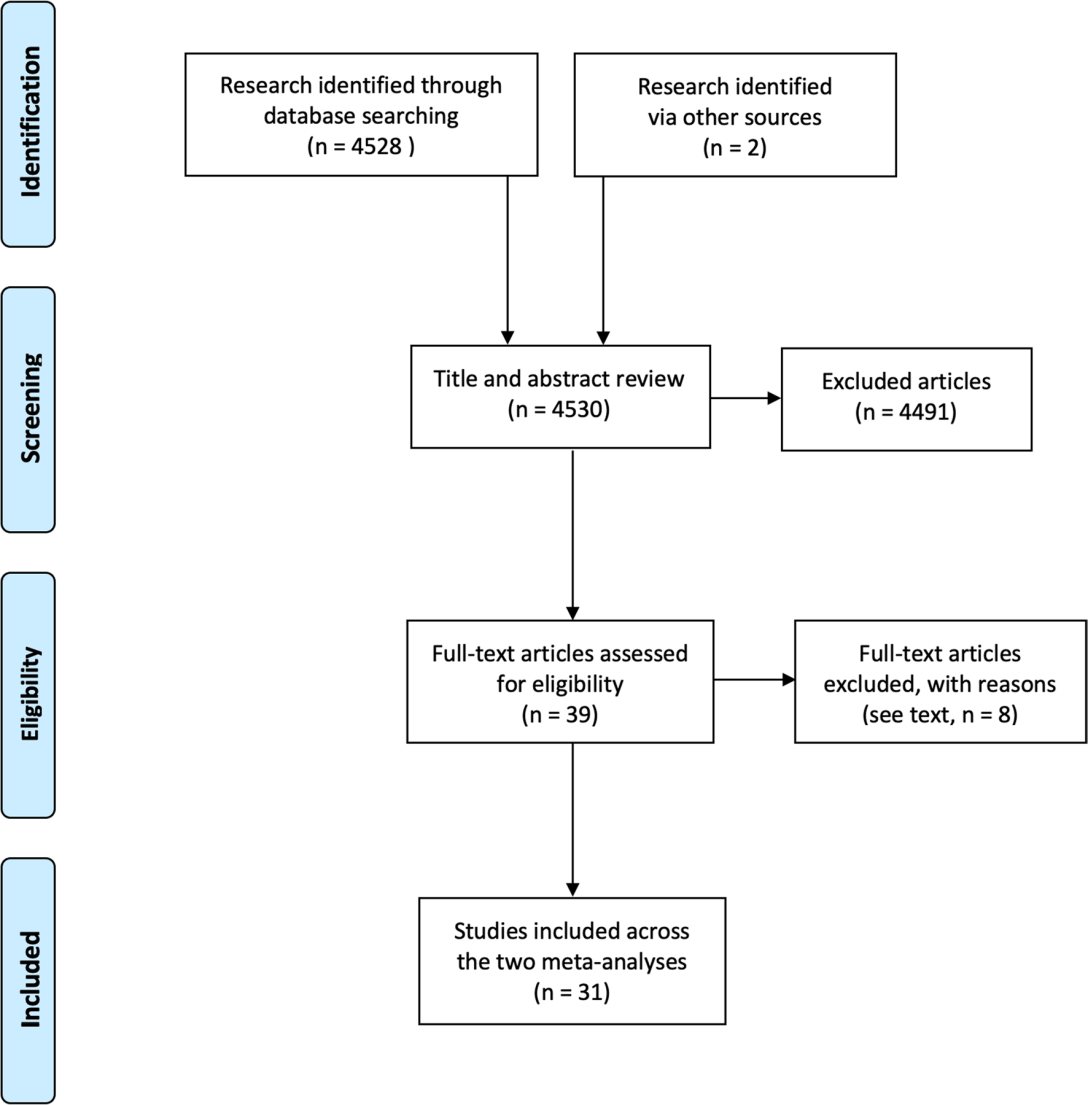
Study PRISMA flowchart

The PRISMA flowchart is presented in Fig. 1. The literature search identified 4530 potential articles. After the title and abstracts review, 39 underwent full-text evaluation, and 8 were excluded for the following reasons: one was a systematic review [15]; two reported insufficient surgical data [16, 17]; three reported laboratory or imaging variables that failed to reach the five article threshold [18–20]; one did not differentiate small from large bowel obstruction [21]; and one with excessive selection bias [22]. This left 31 studies for inclusion [4–11, 23–44].

Table 1 describes the characteristics of the included studies. In total, 4638 patients were analysed, with a median sample size of 128, ranging between 44 [28] and 313 [23]. Mean patient age per-study ranged from 52 [9] to 73 years [28]. The male-to-female ratio was approximately equal across most studies, except one that reported 77% female participants [28]. Most studies (29, 94%) were single centre, with one study conducted across three USA hospitals [7] and one across four French hospitals [25]. Most studies (23, 74%) gathered data retrospectively via case-note review. Seven were prospective cohort studies [7, 11, 29, 33, 34, 39, 41]. Only one study was a randomised controlled trial [26].

**Table 1:** Characteristics of studies included in the review, including raw data for patients treated conservatively versus surgically and those with and without small bowel ischaemia at surgery

Study	Dates	Setting	Study design	Imaging modality	Analysis method	Sample size	Number of episodes	Mean age	Median age	Surgery	Ischaemia
Assenza 2016*	2005–2014	Italy	Retrospective	AXR + CT	UV	313		64		225	42
Bouassida 2020	2008–2017	Tunisia	Retrospective	CT	UV + MV + MDev	124			52	89	23
Cengel 2021	2015–2019	Turkey	Retrospective	CT	UV + MV	228		55		76	7
Chang 2014	2006–2011	Taiwan	Retrospective	CT	UV + MV + MDev	151		62		63	
Cosse 2013	2006–2009	France	RCT	CT	UV + MV	166		61		35	
Ferris 2021	2017	Australia	Retrospective	CT	UV	81	82	67		39	15
Geffroy 2014	2006–2009	France	Retrospective	CT	UV + MV	44		73			19
Hwang 2009	2005–2008	South Korea	Prospective	CT	UV	128		56		37	
Jancelewicz 2008	1996–2006	USA	Retrospective	CT	UV + MV	192					44
Jones 2007	2004–2005	USA	Retrospective	CT	UV + MV + SDev	96		63		53	3
Khaled 2018	2010–2015	France	Retrospective	CT	UV + MV	216	237	71	74	30	21
Kogha 2017	2009–2016	Japan	Retrospective	CT	UV	57					30
Komatsu 2010	2000–2004	Japan	Retrospective	AXR + CT	UV + MV + MDev	154				25	
Kuehn 2017		Germany	Prospective	AXR + CT	UV	105			63	20	6
Kulvatunyou 2015	2011–2013	USA	Prospective	CT	UV + MV + MDev	200		60		52	
Markogiannakis 2011	2005	Greece	Prospective	AXR + CT	UV + MV	100		64			35
Millet 2014	2006–2012	France	Retrospective	CT	UV + MV	159			69	46	
Millet 2017	2009–2015	France	Retrospective	CT	UV + MV	256			64	105	62
Mu 2018	2013–2016	China	Retrospective	CT	UV + MV	288		55	54	122	37
O’Daly 2009	2002–2004	Ireland	Retrospective	CT	UV + MV	88				30	9
O’Leary 2014	2009	USA	Retrospective	AXR + CT	UV + MV + SDev	219		62	63	77	
O’Leary 2016	2008–2013	USA	Retrospective	CT	UV + MV	116		52		116	35
Perea García 2004	1999–2001	Spain	Prospective	AXR	UV	100		64		25	9
Pricolo 2016		USA	Retrospective	CT	UV	108				18	
Schwenter 2010	2004–2007	Switzerland	Prospective	CT	UV + MV + SDev	221	233		71	138	45
Scrima 2017		USA	Retrospective	CT	UV + MV	179		56		56	10
Suri 2014	2004–2006	Canada	Retrospective	CT	UV + MV	63				27	6
Tanaka 2008	2003–2006	Japan	Retrospective	AXR	UV + MV	53		67		14	
Yang 2017	2009–2015	Australia	Retrospective	CT	UV + MV + MVal	233		68		73	
Zielinski 2010*	2006	USA	Retrospective	CT	UV + MV + MDev	100		64		48	11
Zielinski 2011	2009	USA	Prospective	CT	UV + MV + MVal	100		65	67	51	9

^*^Studies with separate outcomes for surgery and ischaemia

Abbreviations: *UV* univariable, *MV* multivariable, *MDev* model development, *MVal* model validation, *CT* computed tomography, *AXR* abdominal radiography, *RCT* randomised controlled trial

“Number of episodes” refers to individual obstructive episodes reported since some patients suffered multiple obstructive episodes

### Risk of bias

The risk of bias is reported in Online Supplementary Material 2. Only 11 (35%) studies raised no concerns regarding the risk of bias [4, 5, 7, 8, 26, 28, 30, 31, 33, 40, 41]. Analysis revealed an unclear or high risk of bias for patient selection in 9 studies (29%) [9, 23, 24, 27, 32, 35, 38, 39, 43]. Additionally, the risk of bias for the predictor and reference standard domains was high or unclear in 42% and 32% of studies, respectively. The main factors underpinning high or unclear risk of bias were failure to report how outcomes were determined and/or difficulty in understanding whether predictors were interpreted without prior knowledge of the clinical outcome and vice versa. In contrast, applicability scores fared much better, with only one article raising concerns and, even then, only judged “unclear” [23].

### Imaging, clinical, and laboratory predictors

Computed tomography (CT) was the modality most commonly used to assess patients, as reported in 29 (94%) studies (Table 1). In addition, five studies combined CT and abdominal radiography (AXR), while two studies used AXR alone [39, 44]. To simplify data presentation, we merged the different terms used for similar predictors into eight groups; for example, “rebound tenderness,” “guarding,” and “peritonitis” were merged under “peritonism” (Table 2). A total of 29 potential predictor variables were identified for meta-analysis, representing 14 imaging, 10 clinical, and 5 laboratory variables (Table 3).

**Table 2:** Description of merged terms used for the review

Terms used in individual studies	Merged terms used in systematic review
- Rebound tenderness (3) - Guarding (3) - Peritonitis (4)	Peritonism (signs of)
- Constipation - Absence of flatus - Bowel not opened	Obstipation
- Free fluid - Ascites	Peritoneal free fluid
- Cardiac disease - Vascular disease - Hypertension (n = 2)	Cardiovascular disease
- Mesenteric congestion - Mesenteric oedema - Mesenteric haziness - Mesenteric fluid - Mesenteric stranding	Mesenteric inflammatory changes
- U- or C-shaped bowel - Multiple transition points - Beak sign	Closed loop
- Pneumatosis intestinalis (6) - Portal venous gas (4) - Mesenteric venous gas (2) - Intramural gas (2)	Signs of bowel wall necrosis
- Grade of obstruction (4) - Degree of obstruction (5)	Degree of obstruction

Numbers in brackets refer to the number of original studies in which the term was used

**Table 3:** Potential imaging, clinical, and laboratory predictor variables extracted for the review

Clinical	Laboratory	Radiological
Prior history of SBO	White blood count (WBC)	Thickened bowel
History of abdominal or pelvic surgery	Creatine (mg/dl)	Dilated small bowel
Peritonism	Blood urea nitrogen (BUN, mg/dl)	Decreased bowel wall enhancement
Tachycardia	Lactate (mmol/l)	Presence of a transition point
Abdominal distension	C-reactive protein (CRP, mg/l)	Closed loop
Pain		Faeces sign
Fever		Grade of obstruction (Low or high)
Obstipation		Presence of peritoneal free fluid
Nausea and/or vomiting		Mesenteric inflammatory changes
History of cardiac and/or vascular disease		Signs of bowel necrosis
		Pneumoperitoneum
		Air-fluid level
		Whirl sign
		Presence of contrast in the colon

### Predictive score/model

Eight studies (26%) proposed a predictive model, with three of these describing a risk scoring system. Five of these studies used a combination of CT findings as predictors, including presence of a transition point, small bowel dilatation, intraperitoneal free fluid, reduced bowel wall enhancement, and the presence of closed-loop obstruction [4, 5, 8, 10, 11]. One study included a clinical variable (absence of flatus [7]) and another included a laboratory variable (hyponatraemia [9]) in addition to CT findings. A third proposed a combination of age, nasogastric aspirate volume, and the presence of free fluid on CT to stratify participants into high- and low-risk surgical groups [6].

### Meta-analysis: surgery versus conservative management

**Fig. 2:**
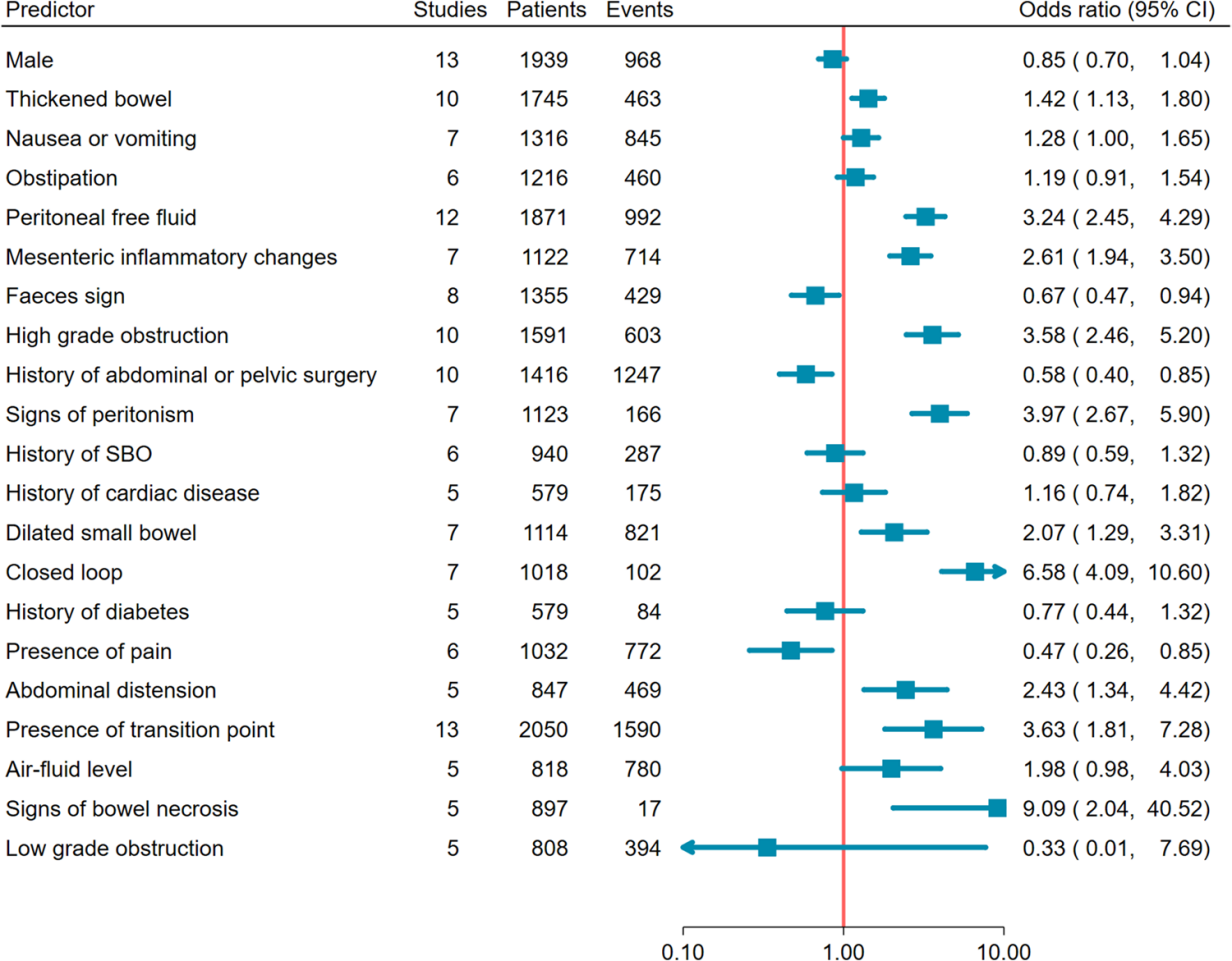
Forest plot of potential imaging, clinical, and laboratory predictor variables reported in more than 5 studies, ordered by confidence interval width. Increasing odds ratio (OR) favours surgery and decreasing OR, conservative management

The strength of evidence for predicting surgery was evaluated in 23 (74%) studies. Of the 29 potential predictors identified, 21 were reported in at least five studies and were meta-analysed. Figure 2 shows meta-analysis results for all 21 predictors, while Online Supplementary Material 3 shows individual study and meta-analyses for each predictor. Using the approach based on narrow confidence intervals and consistent OR, described under “Analysis,” we identified five predictors where the evidence to predict surgery was “strong.” There were three imaging variables: Peritoneal free fluid (OR 3.24, 95%CI 2.45 to 4.29); high-grade obstruction (OR 3.58, 95%CI 2.46 to 5.20); mesenteric inflammatory changes (OR 2.61, 95%CI 1.94 to 3.50). There were two clinical variables: abdominal distention (OR 2.43, 95%CI 1.34 to 4.42) and peritonism (OR 3.97, 95%CI 2.67 to 5.90). Previous abdominopelvic surgery was a strong predictor of conservative management (OR 0.58, 95%CI 0.40 to 0.85).

### Meta-analysis: ischaemia versus no ischaemia

**Fig. 3:**
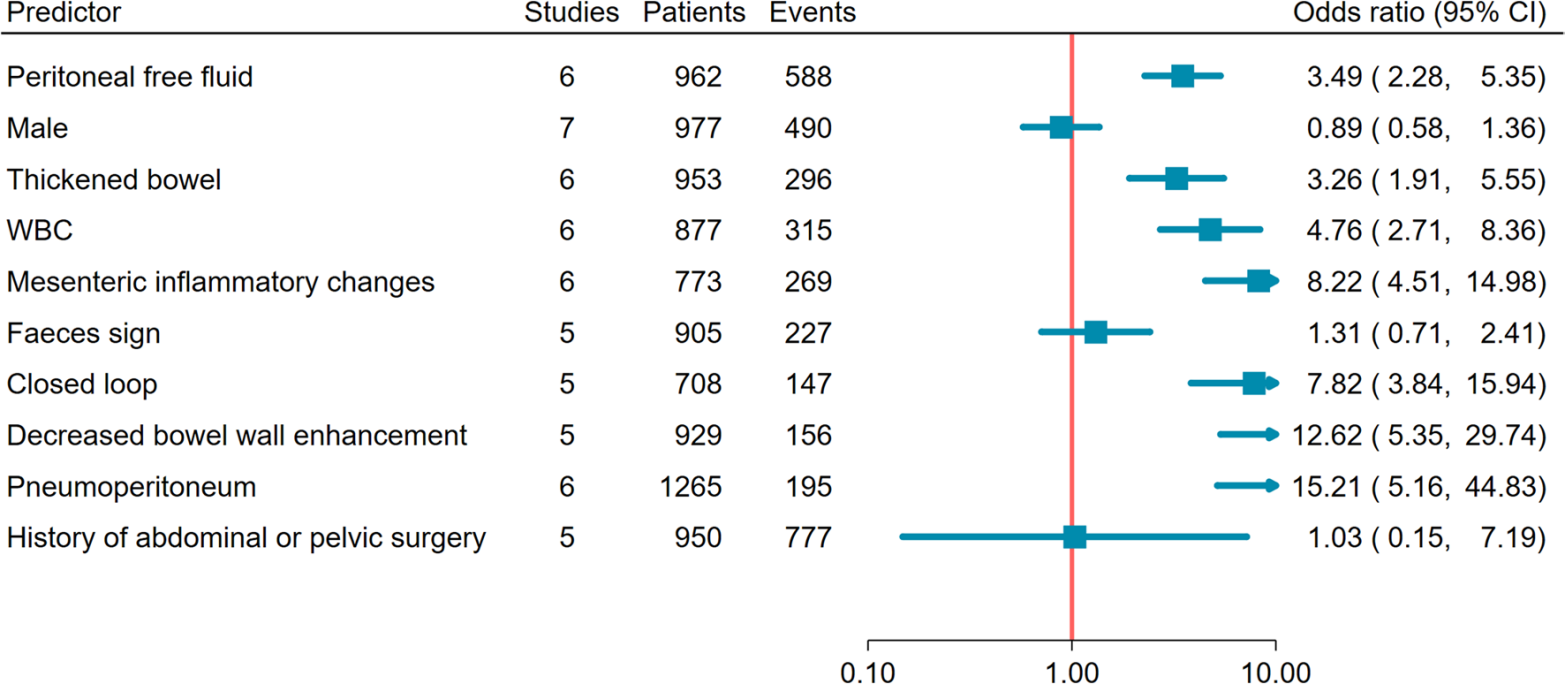
Forest plot of potential imaging, clinical, and laboratory predictor variables reported in more than 5 studies, ordered by confidence interval width. Increasing odds ratio (OR) favours ischaemia identified at surgery and decreasing OR, no ischaemia

The strength of evidence for predicting ischaemic small bowel at surgery was evaluated in ten (32%) studies. Of the 29 potential predictors identified, 10 were reported in at least five studies and were meta-analysed. Figure 3 shows meta-analysis across all 10 predictors while Online Supplementary Material 4 shows individual study metanalyses for each predictor. We identified three predictors where evidence to predict small bowel ischaemia at surgery appeared strong. There were two imaging variables: Peritoneal free fluid (OR 3.49, 95%CI 2.28 to 5.35) and bowel (mural) thickening (OR 3.26 95%CI 1.91 to 5.55). There was one laboratory variable (elevated WBC, OR 4.76, 95%CI 2.71 to 8.36). WBC used various thresholds across studies: ≥ 10 [32, 36], > 10 [24, 38], > 10.5 [10], > 12 [30]. We identified no predictors where evidence to exclude small bowel ischaemia at surgery appeared strong.

## Discussion

A 2015 meta-analysis investigated CT findings that predicted small bowel ischaemia at subsequent surgery for small bowel obstruction [15]. Analysis of 768 patients from nine studies found that reduced mural enhancement was associated with surgical ischaemia, whereas the absence of mesenteric fluid effectively ruled out strangulation [15]. However, it is unlikely that surgeons will restrict their decision-making to imaging alone. Because of this, we performed a systematic review of potential predictor variables that extended beyond CT, into clinical and biochemical realms. In addition to the meta-analysis of factors to predict ischaemia at surgery, we also looked for associations predicting conservative versus operative management. Furthermore, we did not simply rely on statistical significance following meta-analysis to identify promising predictors but based our selection on the strength of statistical evidence, including assessments of consistency and credibility (with non-credible results reflected by excessive OR values and/or excessively wide confidence intervals).

We meta-analysed 14 imaging, 10 clinical, and 5 laboratory predictor variables but could only identify strong evidence of potential utility for 8 of these. Four were imaging variables derived from CT. Peritoneal free fluid, mesenteric inflammation, and high-grade obstruction (defined by clinical suspicion of complete or nearly complete obstruction) all predicted surgical management, whereas peritoneal free fluid and bowel thickening predicted ischaemia at surgery. Three clinical variables demonstrated potential utility to distinguish surgical from conservative management: Abdominal distension and peritonism predicted surgery, whereas a history of previous abdominopelvic surgery predicted conservative management. No clinical variable appeared predictive of ischaemia at surgery, either positively or negatively. Elevated WBC was the only non-imaging variable that appeared useful in that scenario and was the only promising laboratory predictor overall. The fact that Millet’s analysis [15] was restricted to surgical ischaemia may explain why we identified more predictors. Patients ultimately undergoing surgery are a small proportion of those presenting with obstruction, and the fact that study outcomes were more frequently expressed as surgery vs. conservative management (vs. surgical ischaemia or not), presented us with a greater selection of primary research. It should also be noted that many predictor variables were undefined by the authors. For example, “peritoneal free fluid” or “ascites” was not quantified, and of the six studies meta-analysed for “bowel thickening,” only two defined this, and those definitions differed, being “more than 2 mm” in one study [28] versus “greater than 3mm” in the other [8].

Management of SBO remains problematic. A 2018 review stated that “there has been no reliable clinical method for predicting failure of nonoperative management for adhesive SBO” [45]. Accordingly, the primary aim of our review was to identify both imaging and non-imaging factors that should be investigated for the development and evaluation of a multi-variable model predicting treatment strategy for small bowel obstruction. Ideally, such a model would identify those patients unlikely to respond to conservative management and therefore at high risk of ischaemia. At the same time, an accurate model could identify those patients destined to resolve conservatively, and thus avoid surgery. Unfortunately, the large majority of existing literature that investigates predictors of surgery and/or ischaemia is single-centre, and therefore potentially suffers from spectrum bias and/or insufficient power. A single-centre study by Scrima and colleagues sensibly included both imaging and non-imaging factors, but their model was not evaluated [42]. Zielinski and co-workers developed a model based predominantly on CT scanning, finding vomiting, free intraperitoneal fluid, mesenteric oedema, and absent “small bowel faeces” sign, predictive of surgery [10]. The authors then evaluated the model prospectively, adding more predictors and removing predictors initially found significant during development [11]. The American Association for Surgery of Trauma has advocated an “anatomic severity schema” for small bowel obstruction that was validated subsequently in 351 patients [46].

Despite this work, no model has been implemented widely, and surgeons still rely on their clinical impression combined with imaging findings, suggesting that the models lack external validity or are difficult to implement in daily practice. We aimed to facilitate model development via meta-analysis, which allows the mathematical synthesis of potential predictor variables investigated across multiple studies and centres [12]. At the same time, it is statistically undesirable to meta-analyse a limited number of studies unless they are very large, and with sufficient outcomes. This drove our a priori decision to limit meta-analysis to variables reported in five or more individual studies. For example, several small single-centre studies have suggested that water-soluble contrast follow-through (WSCFT, usually “Gastrografin”) may not only diagnose the presence and site of small bowel obstruction, but may also have both prognostic and therapeutic implications. A 2016 meta-analysis found that WSCFT reduced subsequent surgery significantly (OR 0.55) and was 92% sensitive and 93% specific for predicting non-operative management [47]. We particularly wished to investigate this variable since it appears to be widely implemented by surgeons in daily practice. However, we were unable to identify sufficient primary research to allow meta-analysis.

Our review does have limitations. We selected articles that investigated potential imaging predictors of surgery, and of ischaemia at surgery. While we also chose to analyse non-imaging variables, any research that investigated these in the absence of an imaging test would have been omitted. This is because, a priori, we hypothesised that it would be highly unlikely for any high-quality research to omit imaging since the narrative literature suggests CT is the single most useful investigation, and CT has a role both for diagnosis and as a reference standard in patients managed conservatively, which will constitute the majority. Restriction to variables examined in five or more individual studies meant that some potential predictors were not meta-analysed; we have explained the statistical assumptions that underpin our decision. Because we found the risk of bias affected a large proportion of primary studies, our findings should be interpreted with caution. While we were careful not to promote variables simply on the basis of statistical significance following meta-analysis, we also wish to draw readers’ attention to the difference between statistical evidence of utility and clinical conviction. Any potential model development should not ignore variables that are used widely, even where statistical support is absent. WSCFT is the obvious example.

In summary, a systematic review and meta-analysis of imaging, clinical, and laboratory variables of patients with small bowel obstruction identified 6 potential predictors associated strongly with the need for surgery (5 positively and 1 negatively; 3 were derived from CT), and 3 associated strongly with ischaemia at surgery (all positively; 2 were derived from CT). The development of future multivariable models to guide the management of small bowel obstruction should concentrate on variables that appear to display strong evidence of potential utility. Factors that have not been investigated sufficiently well in the primary literature but which enjoy considerable clinical support should also be considered.

### Supplementary Information

Below is the link to the electronic supplementary material.

Supplementary file1 (PDF 5539 KB)

## Abbreviations

AXR: Abdominal x-ray
CI: Confidence interval
CT: Computed tomography
OR: Odds ratio
PRISMA: Preferred reporting terms for systematic reviews and meta-analyses
QUADAS: Quality assessment of diagnostic accuracy studies
SBO: Small bowel obstruction
WSCFT: Water soluble contrast follow-through

## Declarations

### Guarantor

The scientific guarantor of this publication is Steve Halligan.

### Statistics and biometry

Two of the authors (Tom Parry and Sue Mallett) are medical statisticians. One author (Steve Halligan) has a PhD in medical statistics.

### Study subjects or cohorts overlap

This is a systematic review; by definition, all of the primary research has been published. In contrast, our secondary research has not.

### Methodology

• retrospective

• not applicable

• performed at one institution

## References

[1] Diamond M, Lee J, LeBedis CA (2019). Small bowel obstruction and ischemia. Radiol Clin North Am.

[2] Zamary K, Spain DA (2020). Small bowel obstruction: the sun also rises?. J Gastrointest Surg.

[3] Megibow AJ, Balthazar EJ, Cho KC, Medwid SW, Birnbaum BA, Noz ME (1991). Bowel obstruction: evaluation with CT. Radiology.

[4] Chang WC, Ko KH, Lin CS (2014). Features on MDCT that predict surgery in patients with adhesive-related small bowel obstruction. PLoS ONE.

[5] Jones K, Mangram AJ, Lebron RA, Nadalo L, Dunn E (2007). Can a computed tomography scoring system predict the need for surgery in small-bowel obstruction?. Am J Surg.

[6] Komatsu I, Tokuda Y, Shimada G, Jacobs JL, Onodera H (2010). Development of a simple model for predicting need for surgery in patients who initially undergo conservative management for adhesive small bowel obstruction. Am J Surg.

[7] Kulvatunyou N, Pandit V, Moutamn S (2015). A multi-institution prospective observational study of small bowel obstruction: clinical and computerized tomography predictors of which patients may require early surgery. J Trauma Acute Care Surg.

[8] Millet I, Boutot D, Faget C (2017). Assessment of strangulation in adhesive small bowel obstruction on the basis of combined CT findings: implications for clinical care. Radiology.

[9] O'Leary MP, Neville AL, Keeley JA, Kim DY, de Virgilio C, Plurad DS (2016). Predictors of ischemic bowel in patients with small bowel obstruction. Am Surg.

[10] Zielinski MD, Eiken PW, Bannon MP (2010). Small bowel obstruction-who needs an operation? A multivariate prediction model. World J Surg.

[11] Zielinski MD, Eiken PW, Heller SF (2011). Prospective, observational validation of a multivariate small-bowel obstruction model to predict the need for operative intervention. J Am Coll Surg.

[12] Halligan S, Menu Y, Mallett S (2021). Why did European Radiology reject my radiomic biomarker paper? How to correctly evaluate imaging biomarkers in a clinical setting. Eur Radiol.

[13] Page MJ, McKenzie JE, Bossuyt PM (2021). The PRISMA 2020 statement: an updated guideline for reporting systematic reviews. PLoS Med.

[14] Whiting PF, Rutjes AW, Westwood ME (2011). QUADAS-2: a revised tool for the quality assessment of diagnostic accuracy studies. Ann Intern Med.

[15] Millet I, Taourel P, Ruyer A, Molinari N (2015). Value of CT findings to predict surgical ischemia in small bowel obstruction: a systematic review and meta-analysis. Eur Radiol.

[16] Cronk DR, Houseworth TP, Cuadrado DG, Herbert GS, McNutt PM, Azarow KS (2006). Intestinal fatty acid binding protein (I-FABP) for the detection of strangulated mechanical small bowel obstruction. Curr Surg.

[17] Yamamoto Y, Miyagawa Y, Kitazawa M (2021). Association of feces sign with prognosis of non-emergency adhesive small bowel obstruction. Asian J Surg.

[18] Jang KM, Min K, Kim MJ (2010). Diagnostic performance of CT in the detection of intestinal ischemia associated with small-bowel obstruction using maximal attenuation of region of interest. AJR Am J Roentgenol.

[19] Murasaki M, Nakanishi T, Kano KI (2020). Point-of-care procalcitonin may predict the need for surgical treatment in patients with small bowel obstruction. Am J Emerg Med.

[20] Yang JJ, Ma YL, Zhang P, Chen HQ, Liu ZH, Qin HL (2011). Histidine decarboxylase is identified as a potential biomarker of intestinal mucosal injury in patients with acute intestinal obstruction. Mol Med.

[21] Payza U, Kayali A, Bilgin S, Karakaya Z, Esad Topal F (2021). When is the right time to take an emergency surgery decision in Mechanical Intestinal Obstruction?. Asian J Surg.

[22] Kittaka H, Akimoto H, Takeshita H (2014). Usefulness of intestinal fatty acid-binding protein in predicting strangulated small bowel obstruction. PLoS ONE.

[23] Assenza M, De Gruttola I, Rossi D, Castaldi S, Falaschi F, Giuliano G (2016). Adhesions small bowel obstruction in emergency setting: conservative or operative treatment?. G Chir.

[24] Bouassida M, Laamiri G, Zribi S (2020). Predicting intestinal ischaemia in patients with adhesive small bowel obstruction: a simple score. World J Surg.

[25] Cengel F, Gurkan O, Dogan SN, Sayar S (2021). Computed tomography findings predicting the need for surgery in cases of small bowel obstruction: emphasis on duodenal distension. J Comput Assist Tomogr.

[26] Cosse C, Regimbeau JM, Fuks D, Mauvais F, Scotte M (2013). Serum procalcitonin for predicting the failure of conservative management and the need for bowel resection in patients with small bowel obstruction. J Am Coll Surg.

[27] Ferris B, Bastian-Jordan M, Fenwick J, Hislop-Jambrich J (2021). Vascular assessment in small bowel obstruction: can CT predict requirement for surgical intervention?. Abdom Radiol (NY).

[28] Geffroy Y, Boulay-Coletta I, Julles MC, Nakache S, Taourel P, Zins M (2014). Increased unenhanced bowel-wall attenuation at multidetector CT is highly specific of ischemia complicating small-bowel obstruction. Radiology.

[29] Hwang JY, Lee JK, Lee JE, Baek SY (2009). Value of multidetector CT in decision making regarding surgery in patients with small-bowel obstruction due to adhesion. Eur Radiol.

[30] Jancelewicz T, Vu LT, Shawo AE, Yeh B, Gasper WJ, Harris HW (2009). Predicting strangulated small bowel obstruction: an old problem revisited. J Gastrointest Surg.

[31] Khaled W, Millet I, Corno L (2018). Clinical relevance of the feces sign in small-bowel obstruction due to adhesions depends on its location. AJR Am J Roentgenol.

[32] Kohga A, Kawabe A, Yajima K (2017). CT value of the intestine is useful predictor for differentiate irreversible ischaemic changes in strangulated ileus. Abdom Radiol (NY).

[33] Kuehn F, Weinrich M, Ehmann S, Kloker K, Pergolini I, Klar E (2017). Defining the need for surgery in small-bowel obstruction. J Gastrointest Surg.

[34] Markogiannakis H, Memos N, Messaris E (2011). Predictive value of procalcitonin for bowel ischemia and necrosis in bowel obstruction. Surgery.

[35] Millet I, Ruyer A, Alili C (2014). Adhesive small-bowel obstruction: value of CT in identifying findings associated with the effectiveness of nonsurgical treatment. Radiology.

[36] Mu JF, Wang Q, Wang SD (2018). Clinical factors associated with intestinal strangulating obstruction and recurrence in adhesive small bowel obstruction: a retrospective study of 288 cases. Medicine (Baltimore).

[37] O’Daly BJ, Ridgway PF, Keenan N (2009). Detected peritoneal fluid in small bowel obstruction is associated with the need for surgical intervention. Can J Surg.

[38] O’Leary EA, Desale SY, Yi WS (2014). Letting the sun set on small bowel obstruction: can a simple risk score tell us when nonoperative care is inappropriate?. Am Surg.

[39] Perea Garcia J, Turegano Fuentes T, Quijada Garcia B (2004). Adhesive small bowel obstruction: predictive value of oral contrast administration on the need for surgery. Rev Esp Enferm Dig.

[40] Pricolo VE, Curley F (2016). CT scan findings do not predict outcome of nonoperative management in small bowel obstruction: retrospective analysis of 108 consecutive patients. Int J Surg.

[41] Schwenter F, Poletti PA, Platon A, Perneger T, Morel P, Gervaz P (2010). Clinicoradiological score for predicting the risk of strangulated small bowel obstruction. Br J Surg.

[42] Scrima A, Lubner MG, King S, Pankratz J, Kennedy G, Pickhardt PJ (2017). Value of MDCT and clinical and laboratory data for predicting the need for surgical intervention in suspected small-bowel obstruction. AJR Am J Roentgenol.

[43] Suri RR, Vora P, Kirby JM, Ruo L (2014). Computed tomography features associated with operative management for nonstrangulating small bowel obstruction. Can J Surg.

[44] Tanaka S, Yamamoto T, Kubota D (2008). Predictive factors for surgical indication in adhesive small bowel obstruction. Am J Surg.

[45] Bower KL, Lollar DI, Williams SL, Adkins FC, Luyimbazi DT, Bower CE (2018). Small Bowel Obstruction. Surg Clin North Am.

[46] Baghdadi YMK, Morris DS, Choudhry AJ (2016). Validation of the anatomic severity score developed by the American Association for the Surgery of Trauma in small bowel obstruction. J Surg Res.

[47] Ceresoli M, Coccolini F, Catena F (2016). Water-soluble contrast agent in adhesive small bowel obstruction: a systematic review and meta-analysis of diagnostic and therapeutic value. Am J Surg.

