# Supplementary material for: Prognostic factors to identify resolution of small bowel obstruction without need for operative management: systematic review
Source: Eur Radiol. 2023 Nov 8;34(6):3861–71. doi: 10.1007/s00330-023-10421-9 (PMC11166786; doi:10.1007/s00330-023-10421-9)

ONLINE SUPPLEMENTARY MATERIAL 1: Search string used for the PubMed database search.

((((intestinal obstruction) OR (intestinal AND obstruction)) OR (bowel obstruction) OR (ileus) OR (bowel AND obstruction))) AND ((predict OR prognos* OR Stratification OR ROC Curve OR Discrimination OR Discriminate OR c- statistic OR c statistic OR area under the curve auc OR Calibration OR Indices OR Algorithm OR Multivariable)) NOT ((review[Publication Type] OR Bibliography[Publication Type] OR Editorial[Publication Type] OR Letter[Publication Type] OR News[Publication Type]))*

ONLINE SUPPLEMENTARY MATERIAL 2: Risk of bias assessment

	RISK OF BIAS					APPLICABILITY		
**STUDY**	Patient selection	Index tests	Reference standard	Flow and timing	Additional questions	Patient selection	Index test	Reference standard
Assenza 2016	😐	☺	☹	☹	☺	☺	☺	😐
Bouassida 2020	☺	☹	☺	☺	☺	☺	☺	☺
Cengel 2021	😐	☹	☺	☺	☺	☺	☺	☺
Chang 2014	☺	☺	☺	☺	☺	☺	☺	☺
Cosse 2013	☺	☺	☺	☺	☺	☺	☺	☺
Ferris 2021	😐	☺	☺	☺	☺	☺	☺	☺
Geffroy 2014	☺	☺	☺	☺	☺	☺	☺	☺
Hwang 2009	☺	☺	☺	☹	☺	☺	☺	☺
Jancelewicz 2008	☺	☺	☺	☺	☺	☺	☺	☺
Jones 2007	☺	☺	☺	☺	☺	☺	☺	☺
Khaled 2018	☺	☺	☺	☺	☺	☺	☺	☺
Kogha 2017	☹	😐	😐	☺	☺	☺	☺	☺
Komatsu 2010	☺	😐	☺	☺	☺	☺	☺	☺
Kuehn 2017	☺	☺	☺	☺	☺	☺	☺	☺
Kulvatunyou 2015	☺	☺	☺	☺	☺	☺	☺	☺
Markogiannakis 2011	☺	☺	☺	☹	☺	☺	☺	☺
Millet 2014	☹	☺	😐	☺	☺	☺	☺	☺
Millet 2017	☺	☺	☺	☺	☺	☺	☺	☺
Mu 2018	☺	😐	☺	☺	☺	☺	☺	☺
O'Daly 2009	☺	😐	☺	☺	☺	☺	☺	☺
O'Leary 2014	☹	😐	😐	☺	☺	☺	☺	☺
O'Leary 2016	😐	😐	☺	☺	☺	☺	☺	☺
Perea Garcia 2004	😐	😐	☹	☺	☺	☺	☺	☺
Pricolo 2016	☺	☺	☺	☺	☺	☺	☺	☺
Schwenter 2010	☺	☺	☺	☺	☺	☺	☺	☺
Scrima 2017	☺	☺	😐	☺	☺	☺	☺	☺
Suri 2014	☹	☺	☺	☺	☺	☺	☺	☺
Tanaka 2008	☺	😐	☹	☺	☺	☺	☺	☺
Yang 2017	☺	☺	☺	😐	☺	☺	☺	☺

Zielinski 2010	☺ (low)	☺ (low)	😐 (unclear)	☺ (low)	☺ (low)	☺ (low)	☺ (low)	☺ (low)
Zielinski 2011	☺ (low)	😐 (unclear)	☺ (low)	☺ (low)	☺ (low)	☺ (low)	☺ (low)	☺ (low)

Key: Risk of bias ☺ (low) = low, 😐 (unclear) = unclear, ☹ (high) = high

ONLINE SUPPLEMENTARY MATERIAL 3: Meta-analysis of potential predictor variables to predict the need for surgery versus conservative therapy in small bowel obstruction.

Male sex:

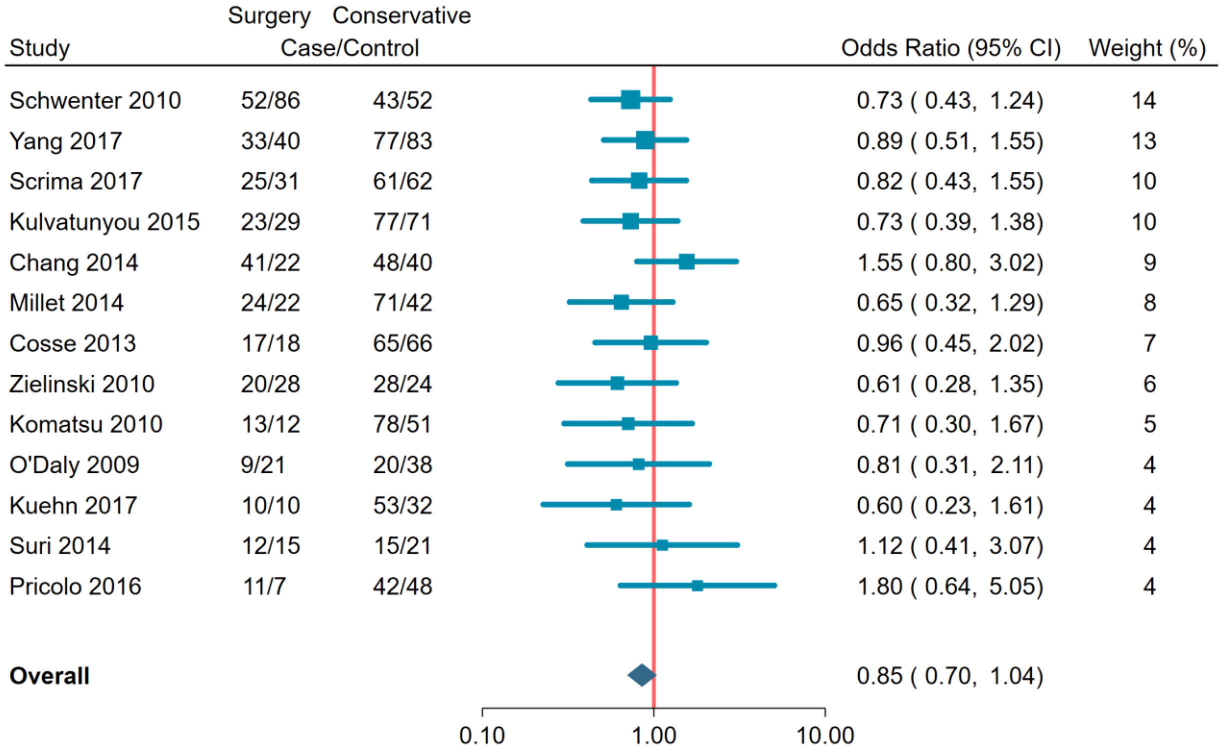

Presence of a transition point:

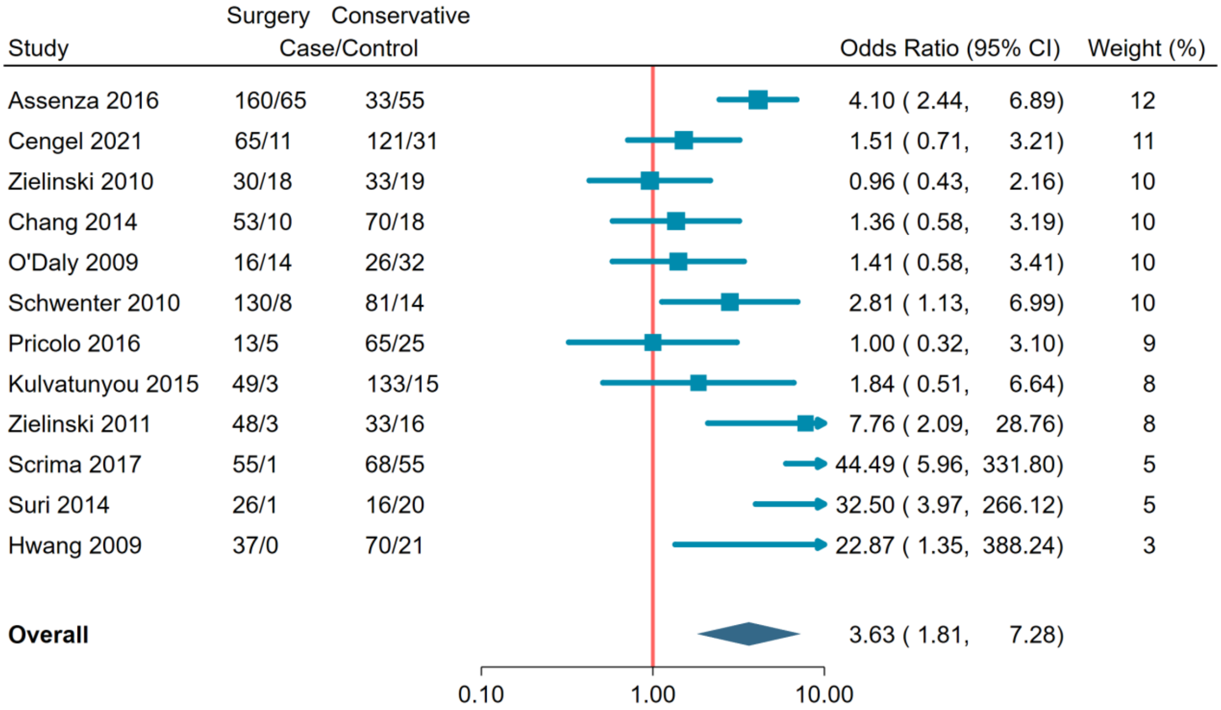

Peritoneal free fluid:

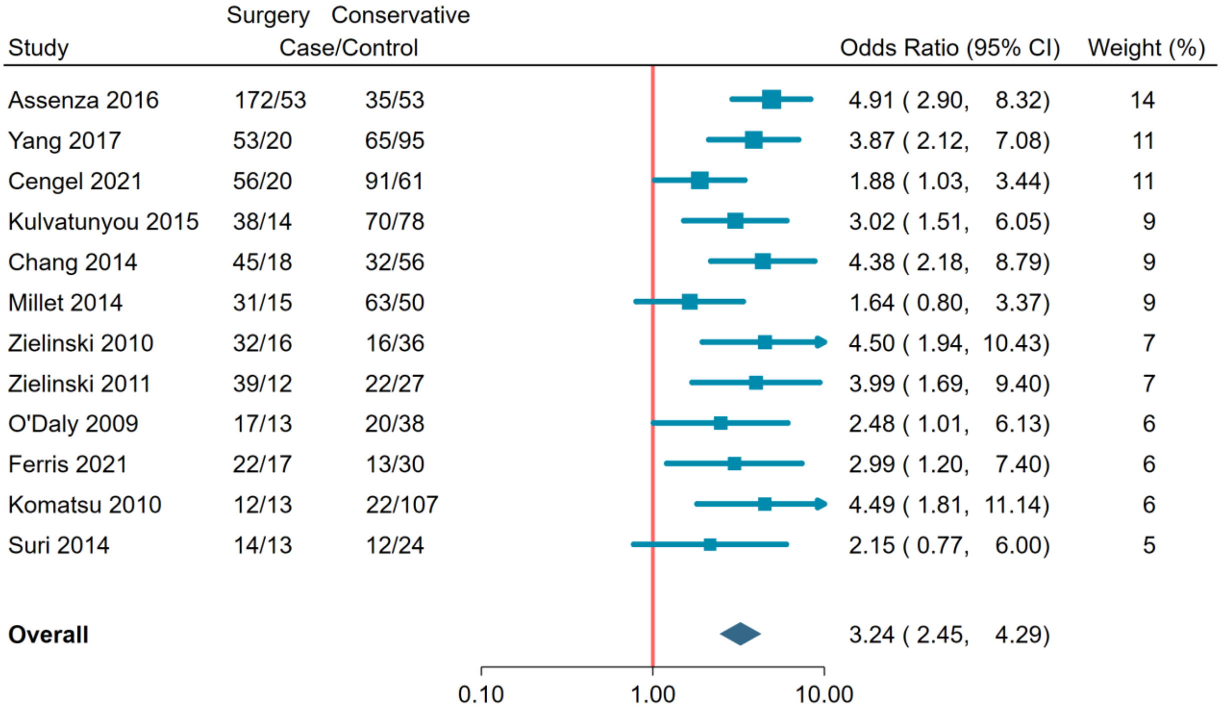

High grade obstruction:

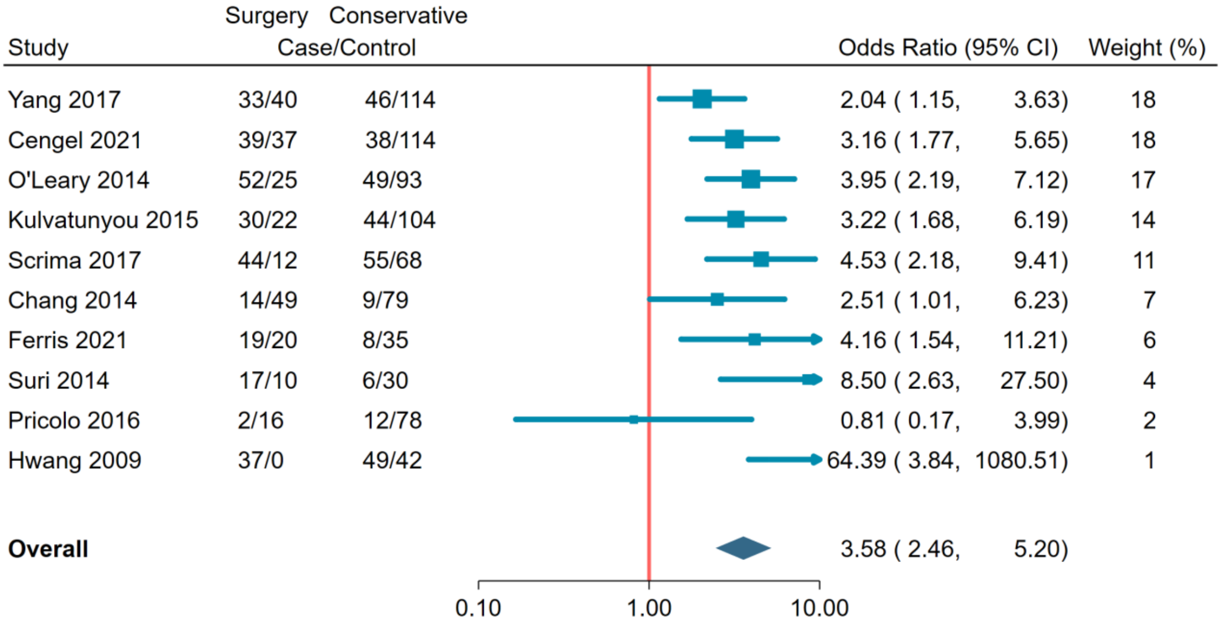

History of abdominal or pelvic surgery:

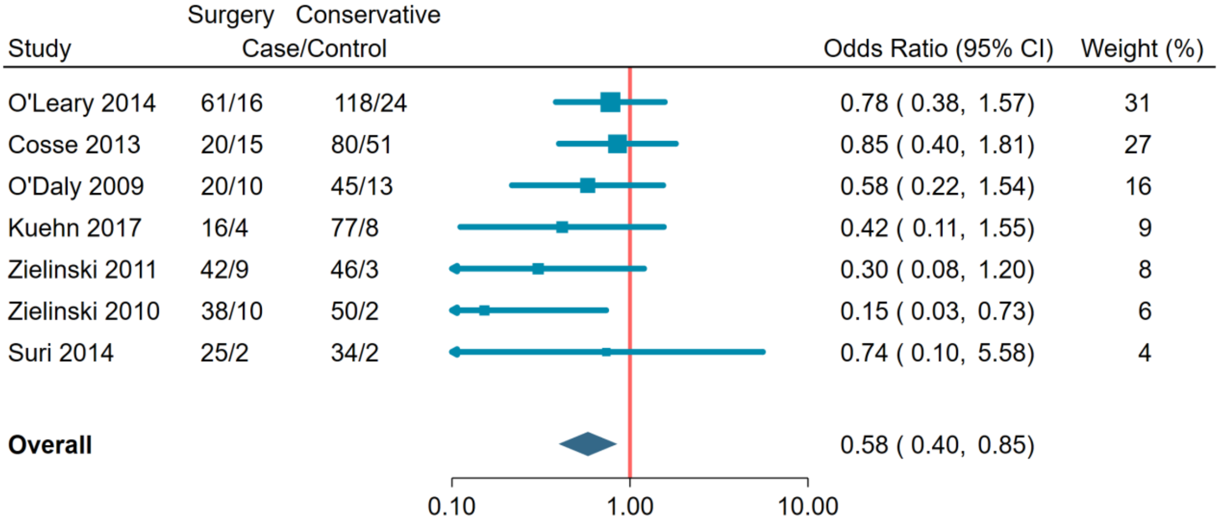

Thickened bowel wall:

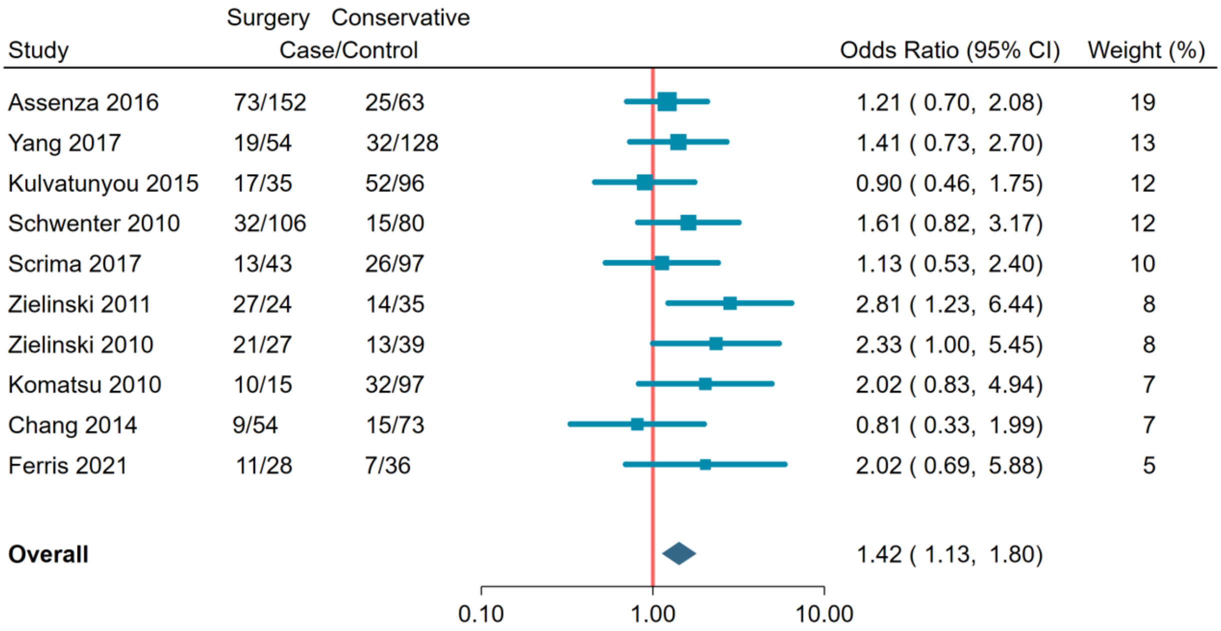

Small bowel faeces sign:

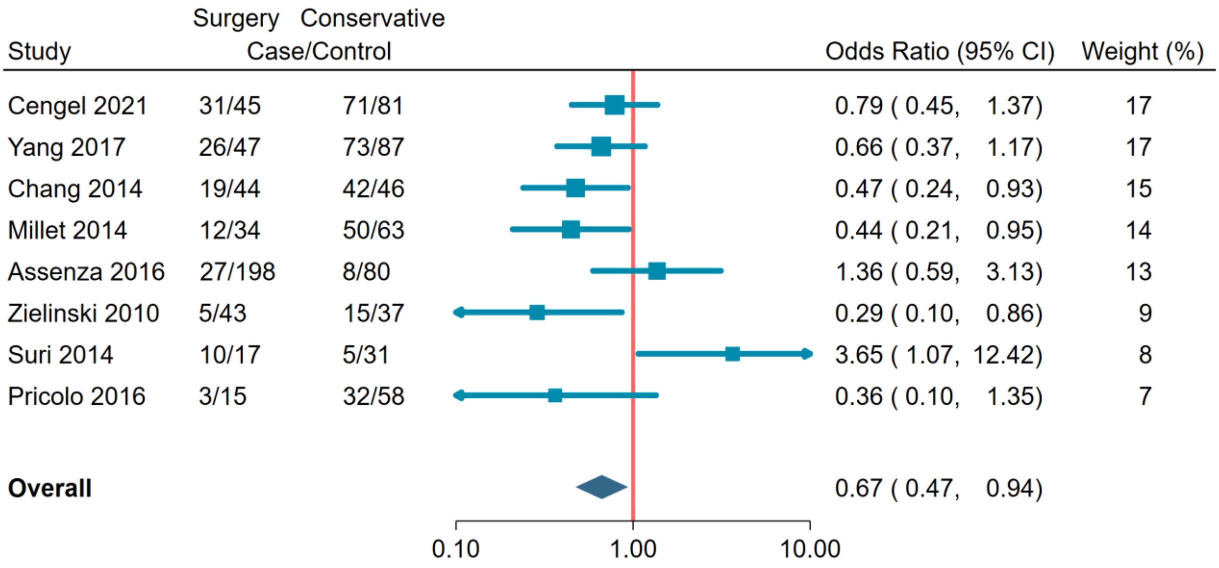

Closed loop obstruction:

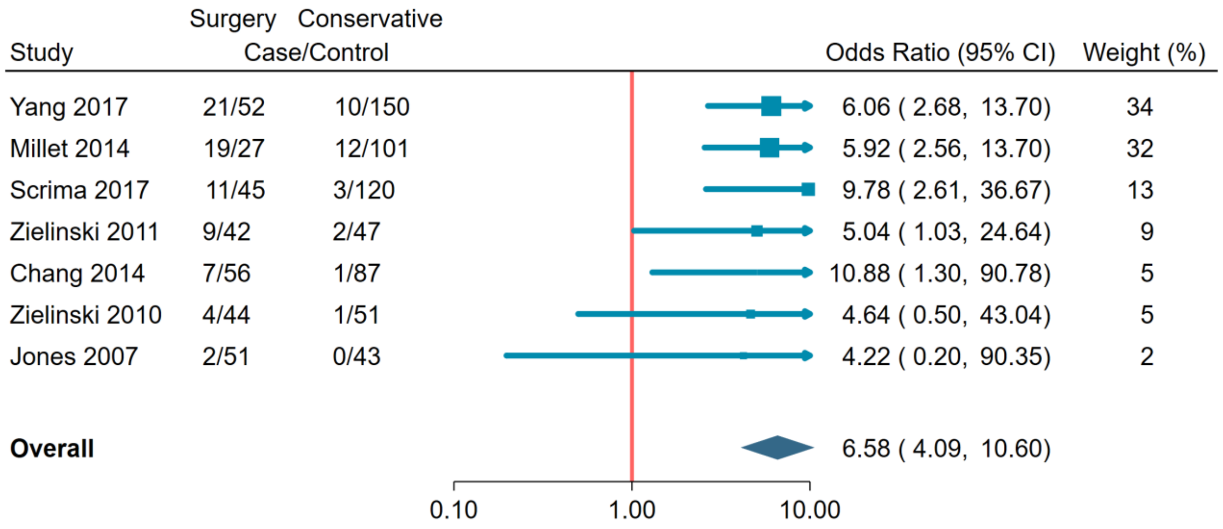

Dilated small bowel:

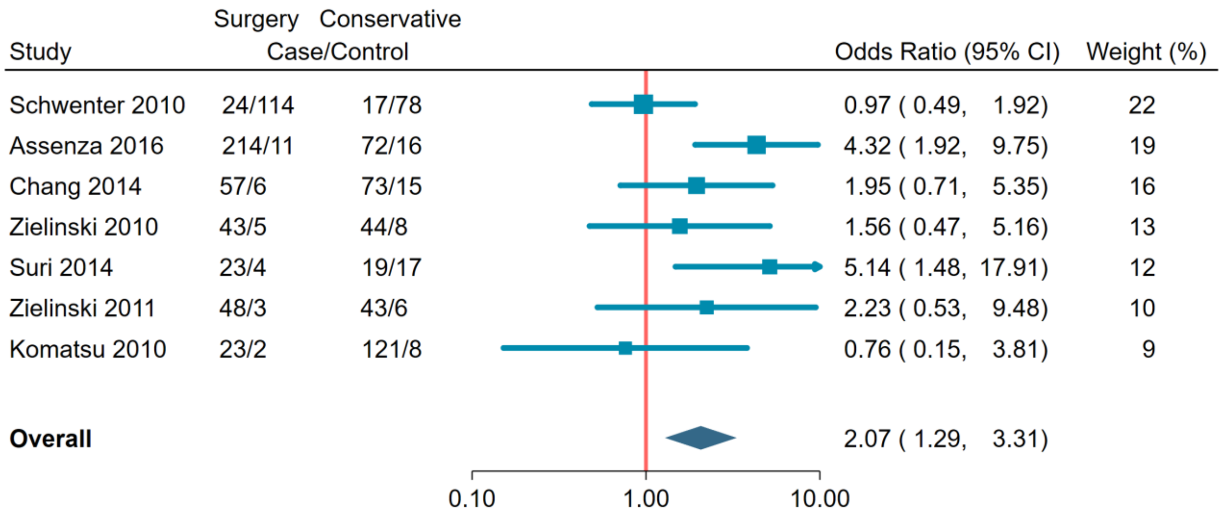

Mesenteric inflammatory changes:

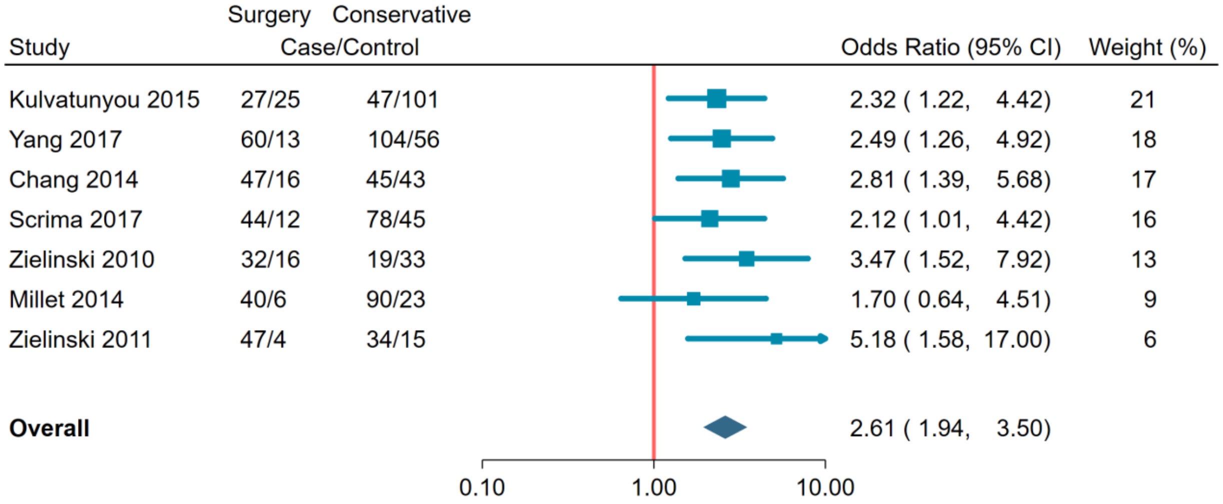

Nausea or vomiting:

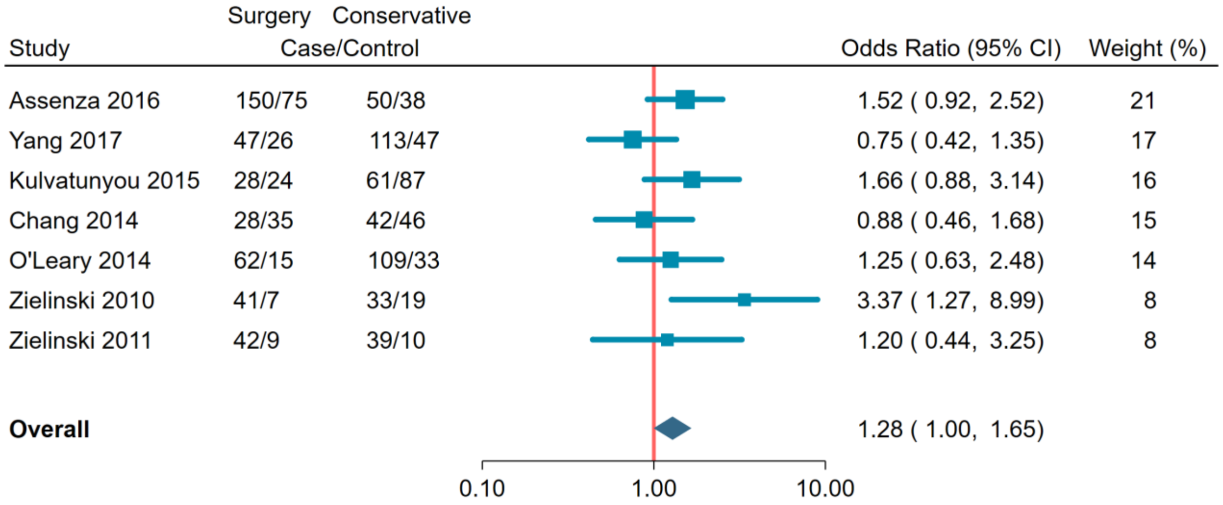

Signs of peritonism:

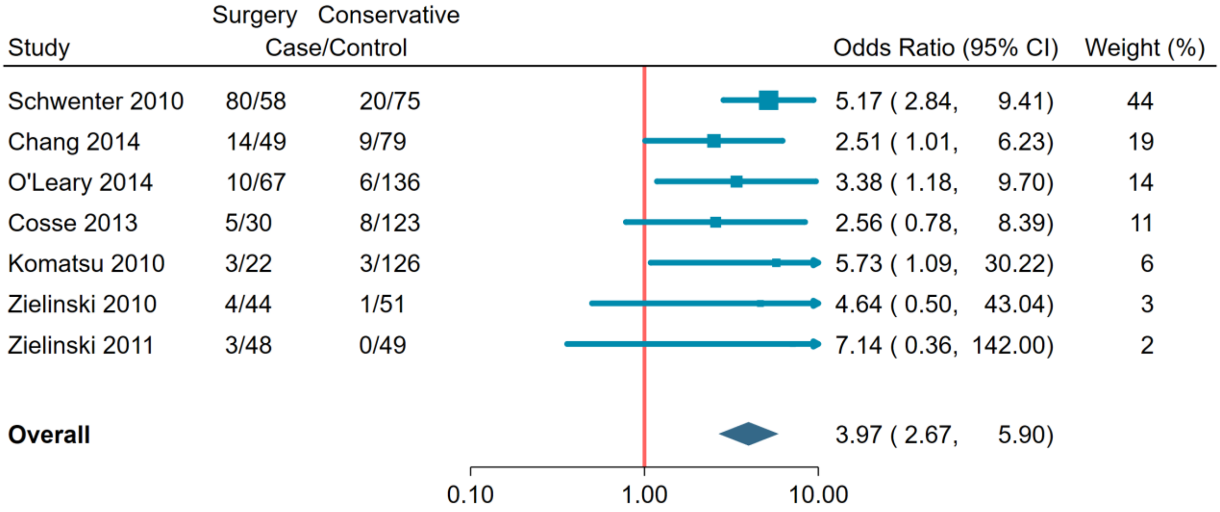

History of small bowel obstruction:

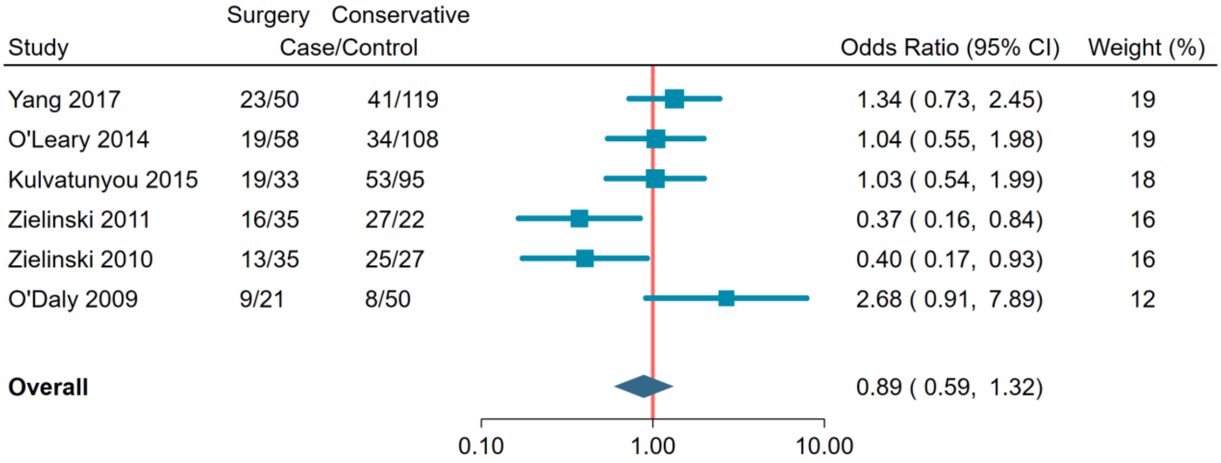

Obstiption:

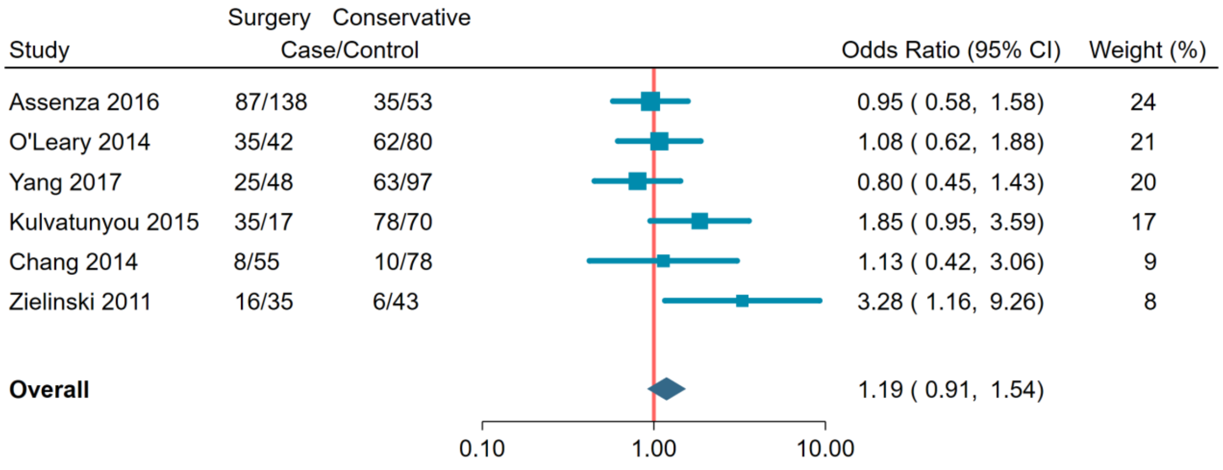

Presence of pain:

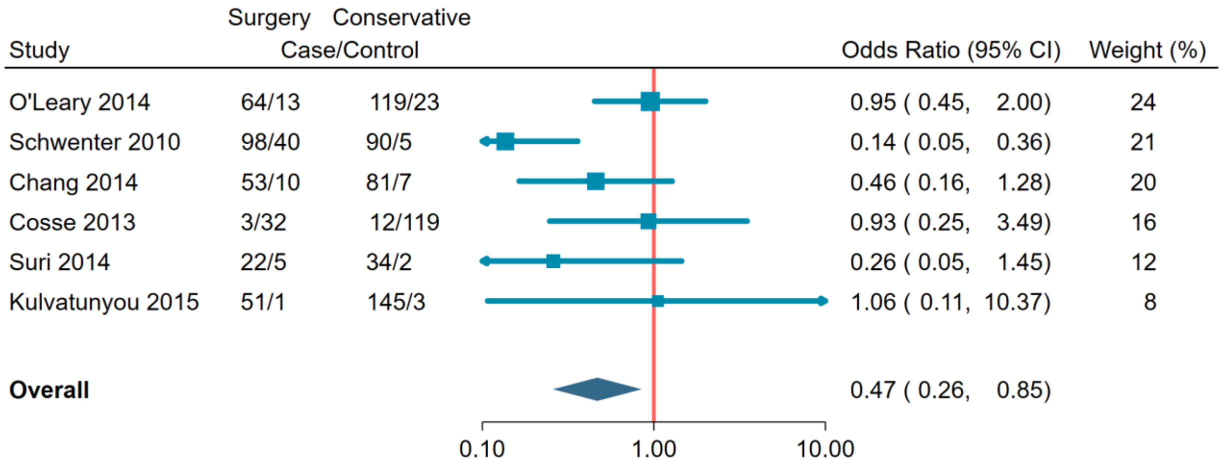

Abdominal distension:

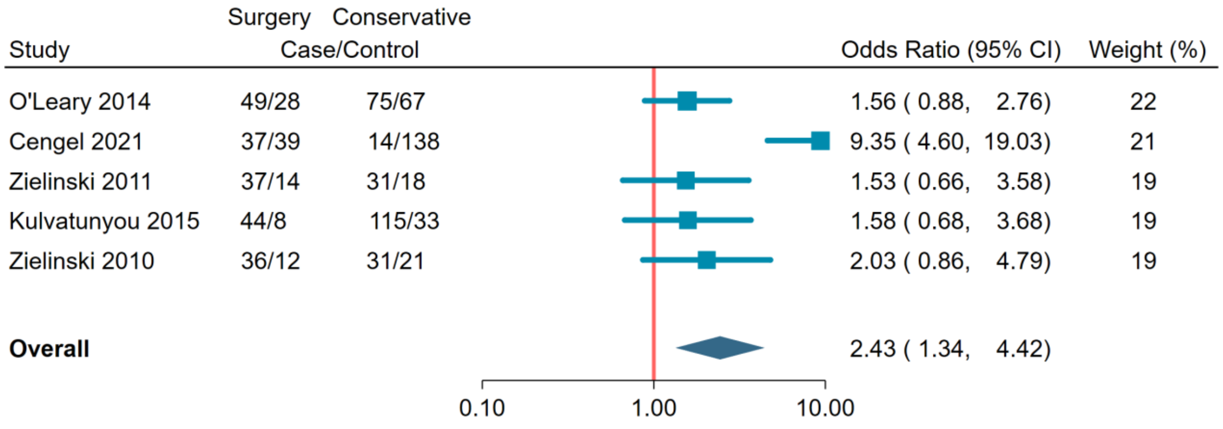

Gas-fluid levels:

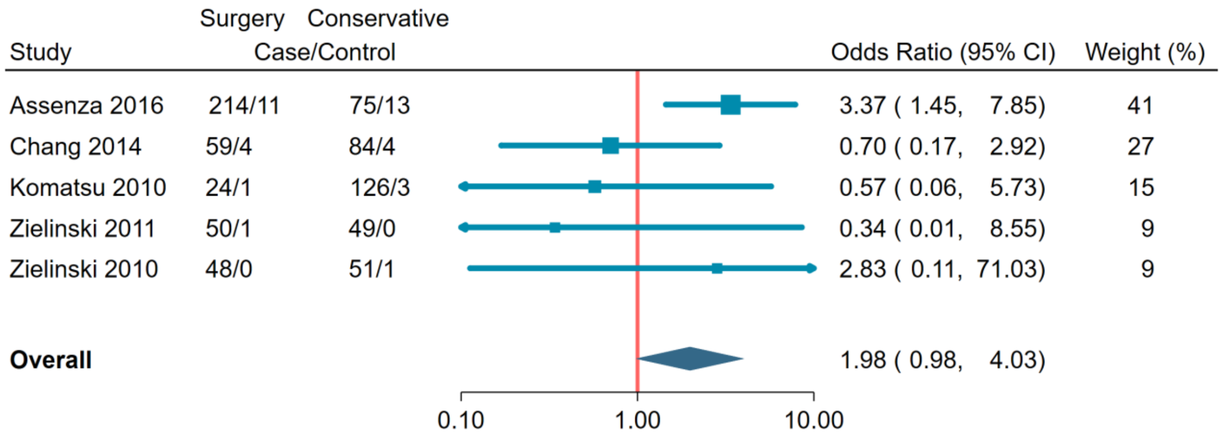

History of cardiac disease:

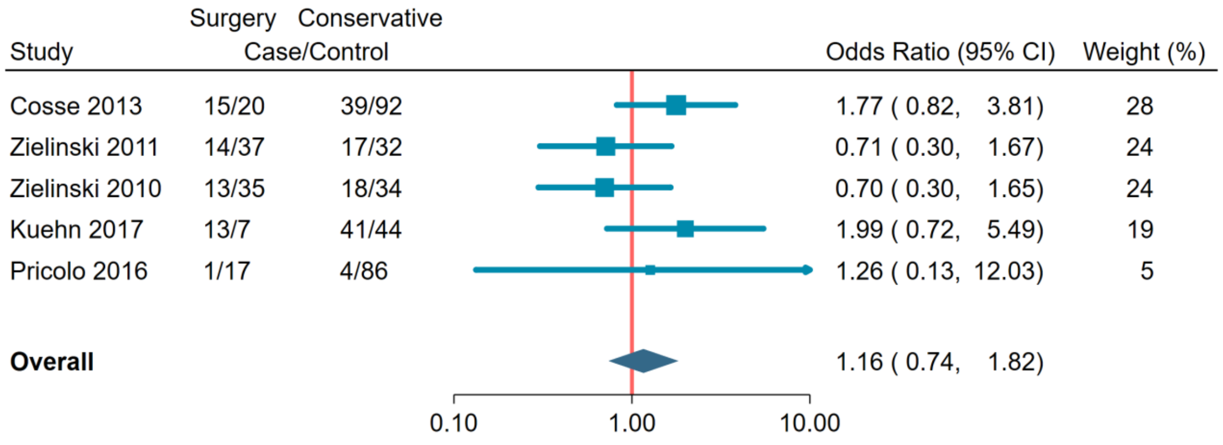

History of diabetes:

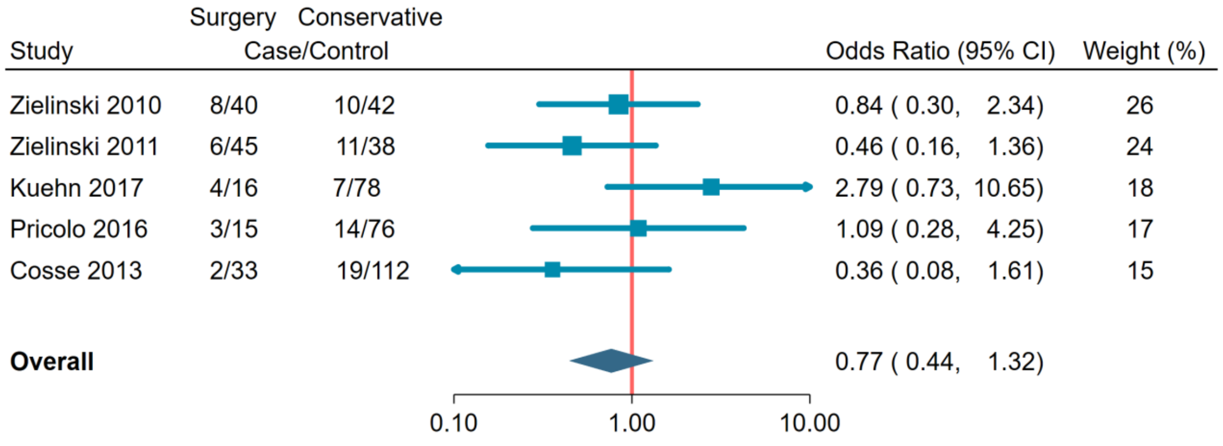

Low grade obstruction:

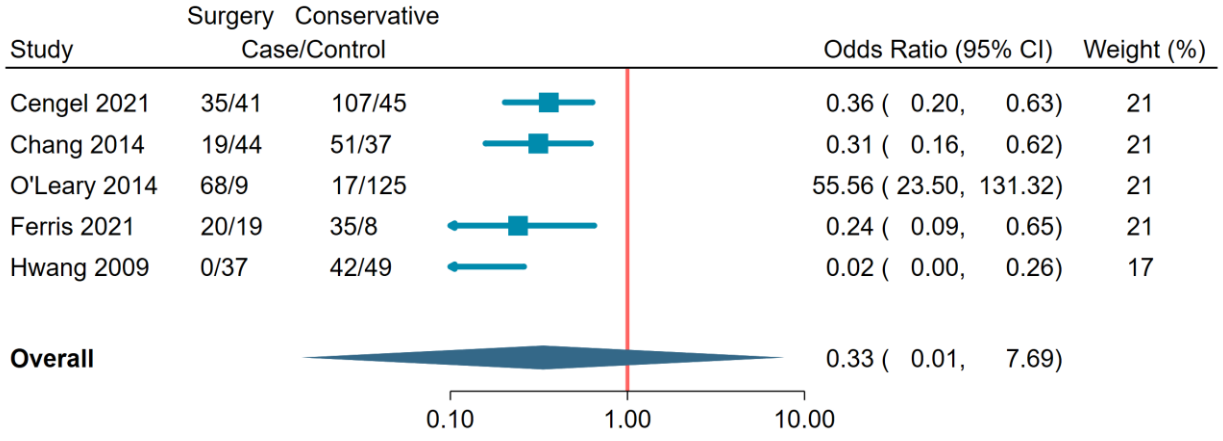

CT signs of bowel necrosis:

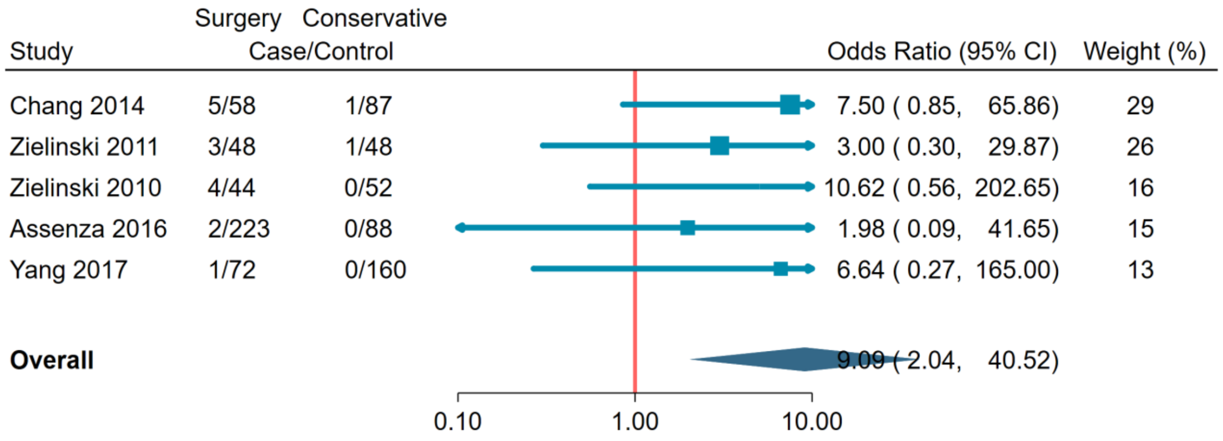

Decreased bowel wall enhancement:

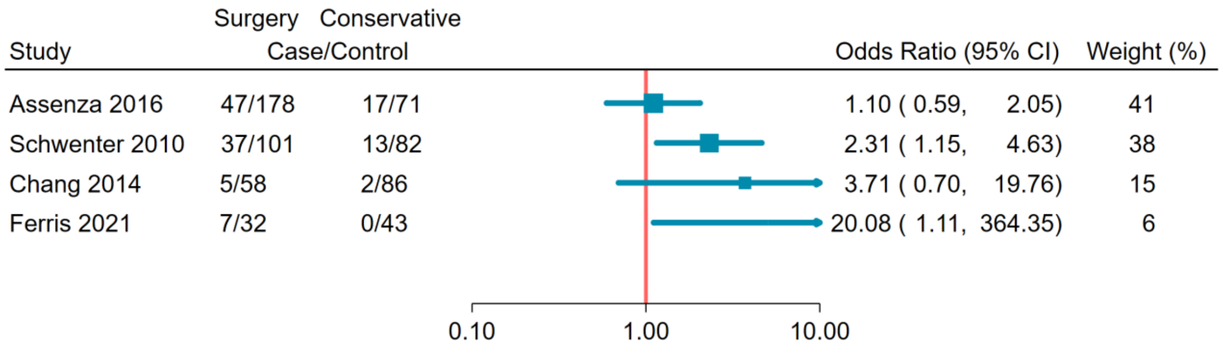

Pyrexia:

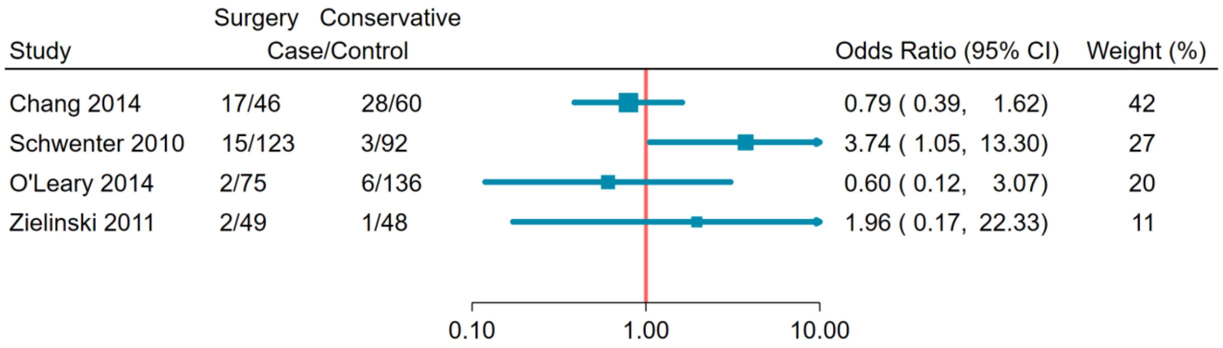

Pneumoperitoneum:

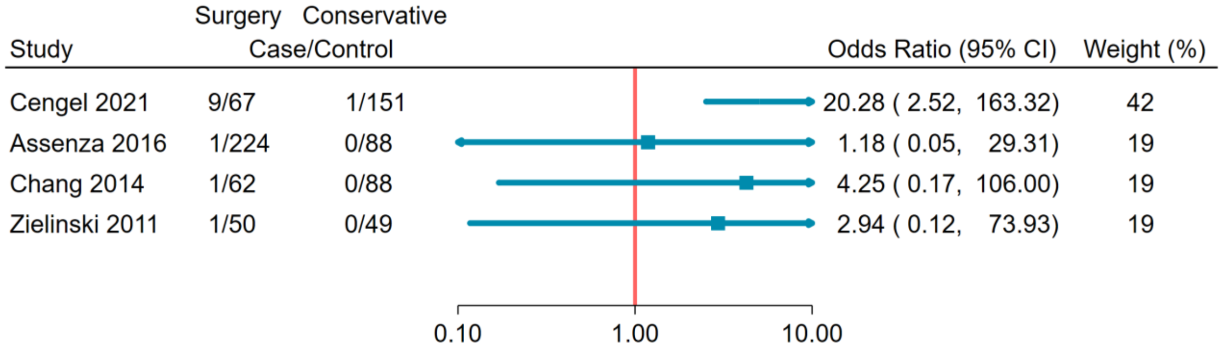

CT whirl sign:

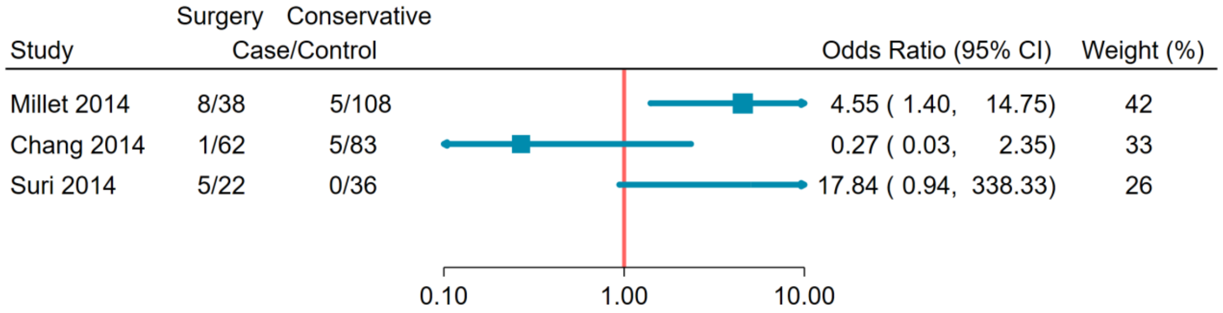

Elevated WBC:

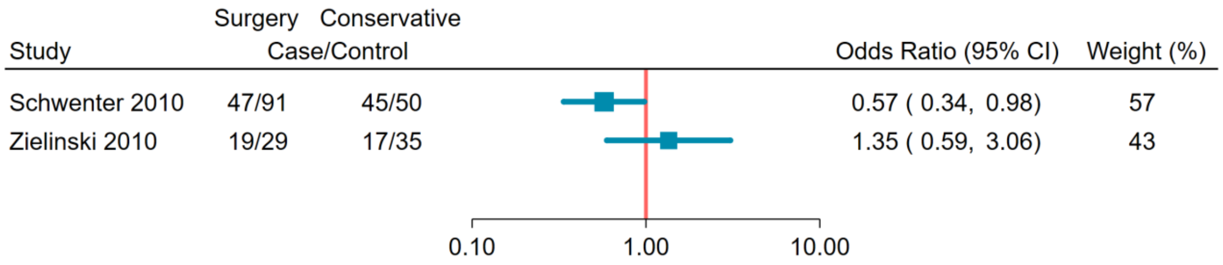

Elevated CRP:

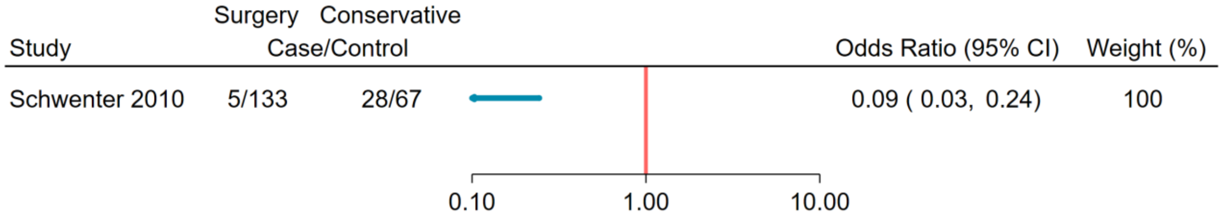

Presence of contrast in the colon:

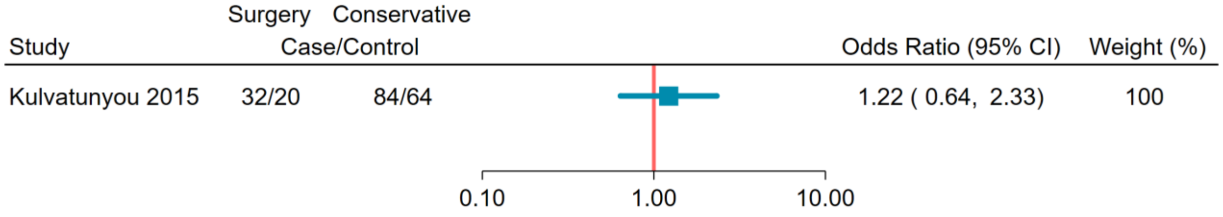

Tachycardia:

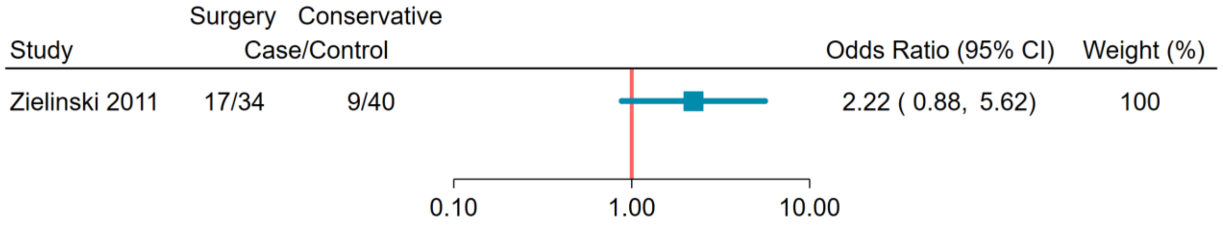

ONLINE SUPPLEMENTARY MATERIAL 4: Meta-analysis of potential predictor variables to predict small bowel ischaemia at surgery for small bowel obstruction:

Male sex:

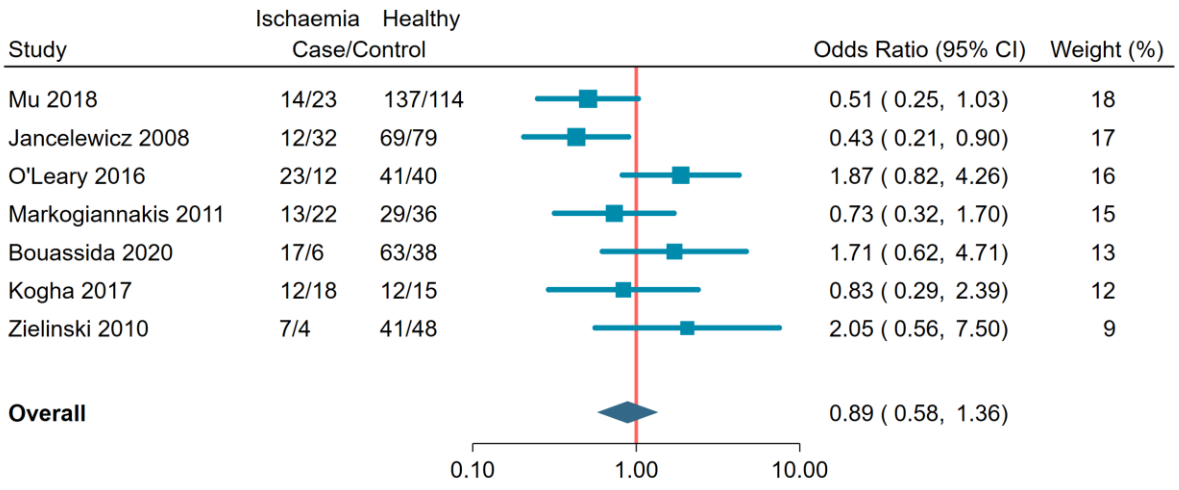

Peritoneal free fluid:

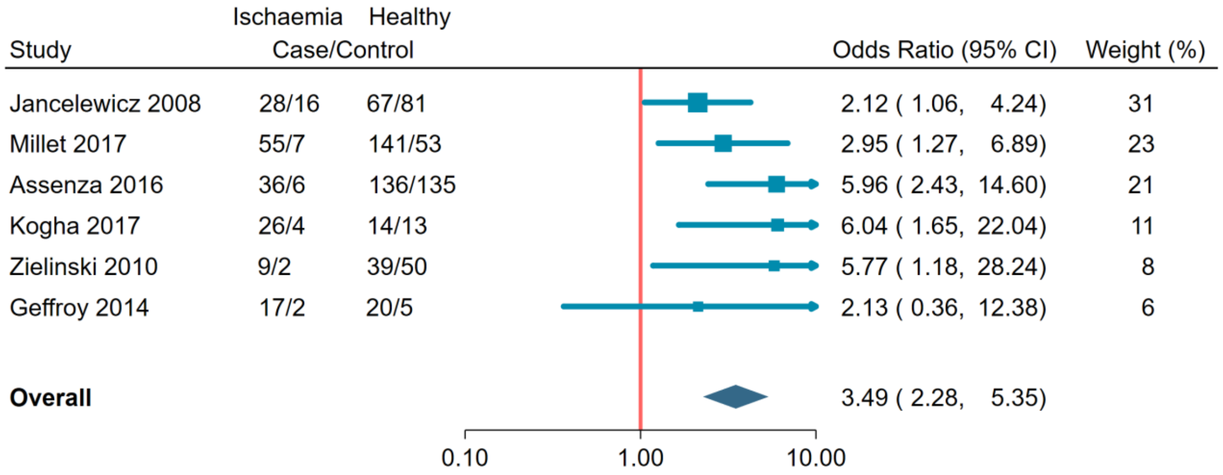

Mesenteric inflammation:

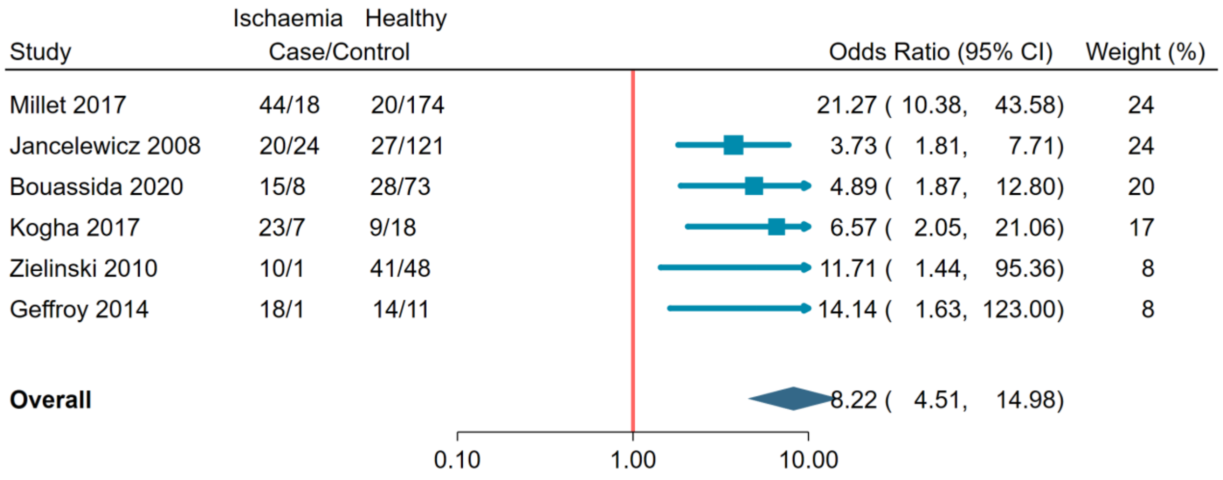

Pneumoperitoneum:

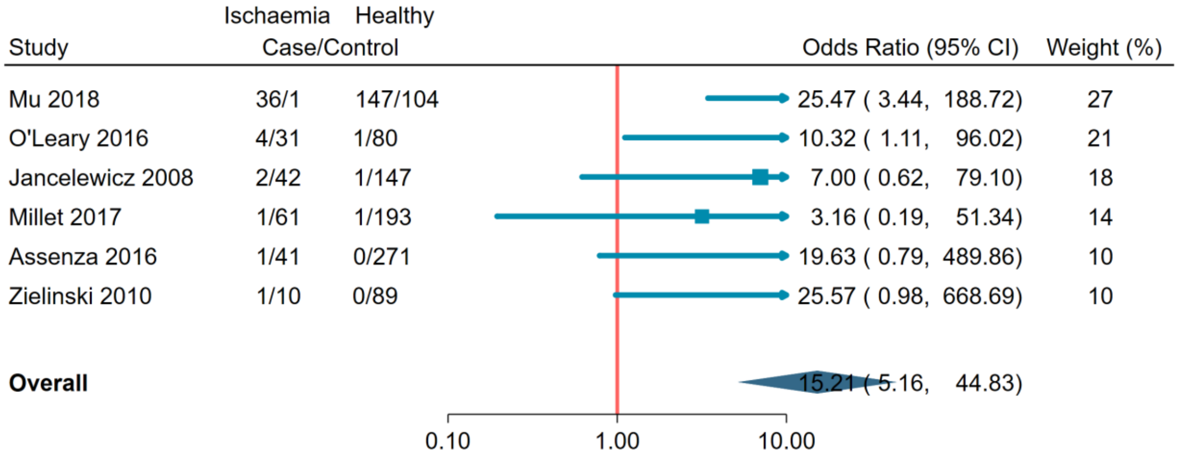

Thickened bowel:

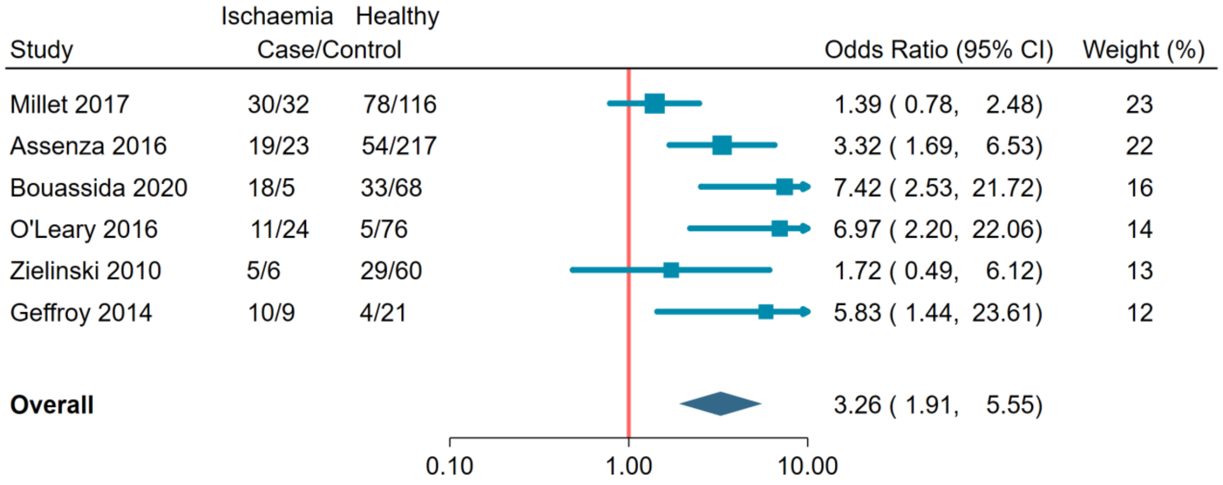

Elevated WBC:

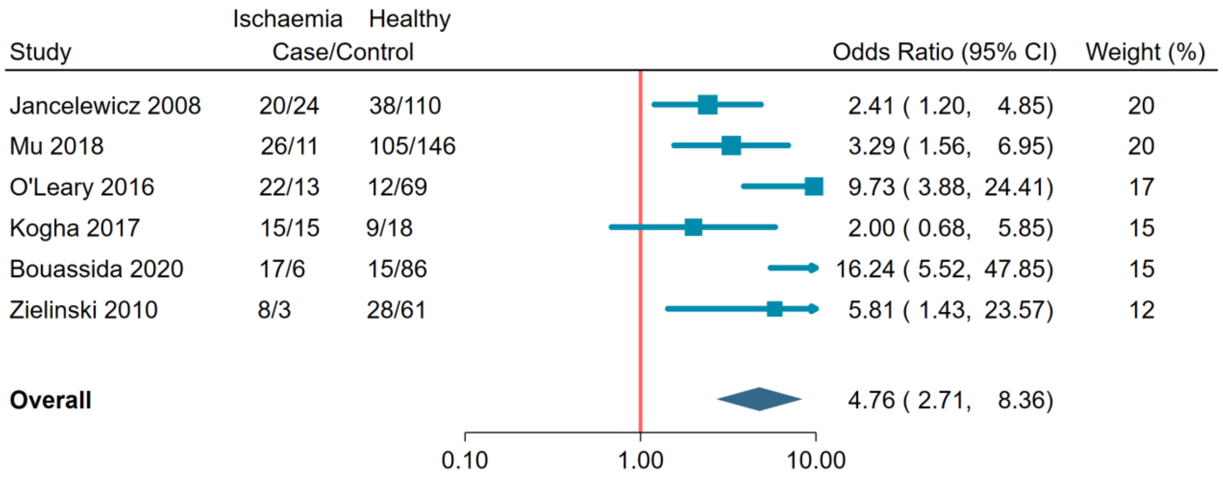

Closed loop obstruction:

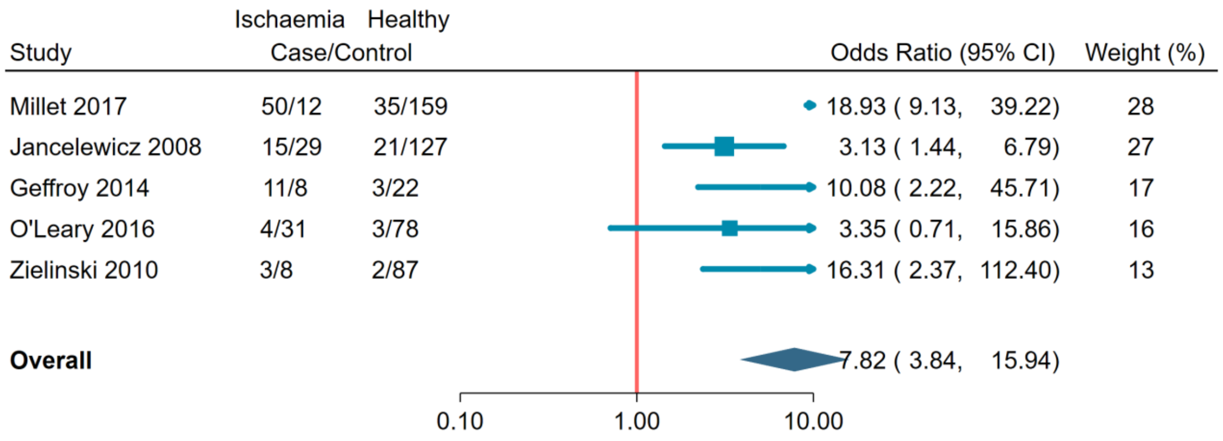

Eur Radiol (2023) Eze VN, Parry T, Boone D, Mallett S, Halligan S

History of abdominopelvic surgery:

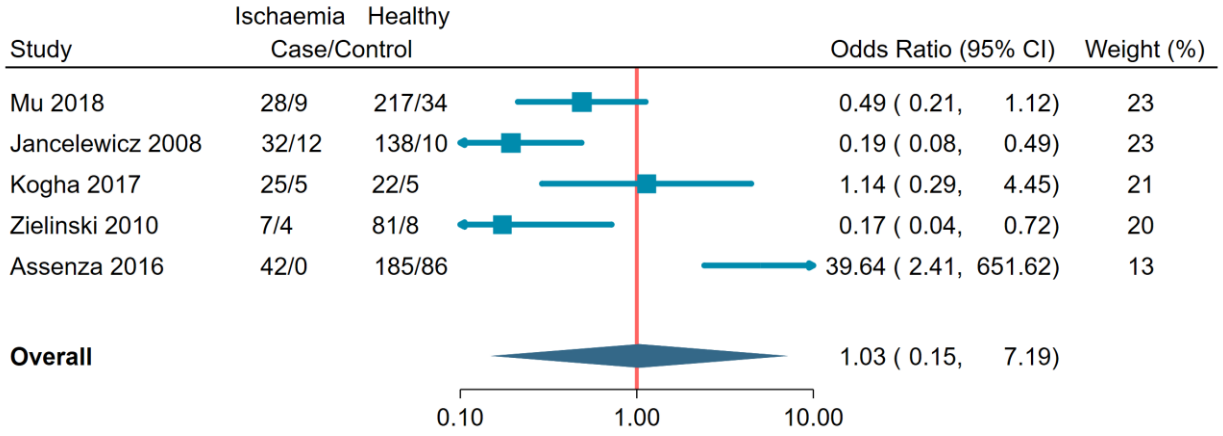

Decreased mural enhancement:

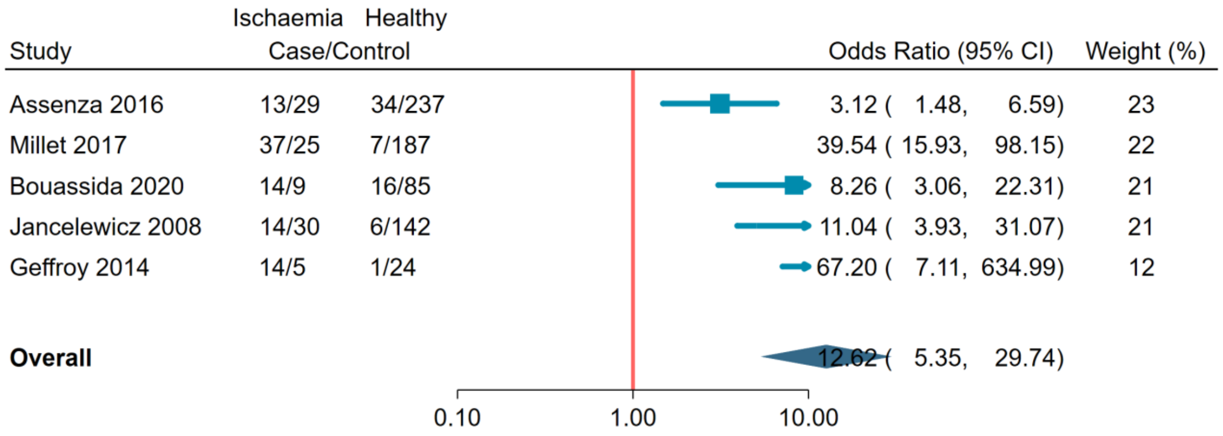

Small bowel faeces sign:

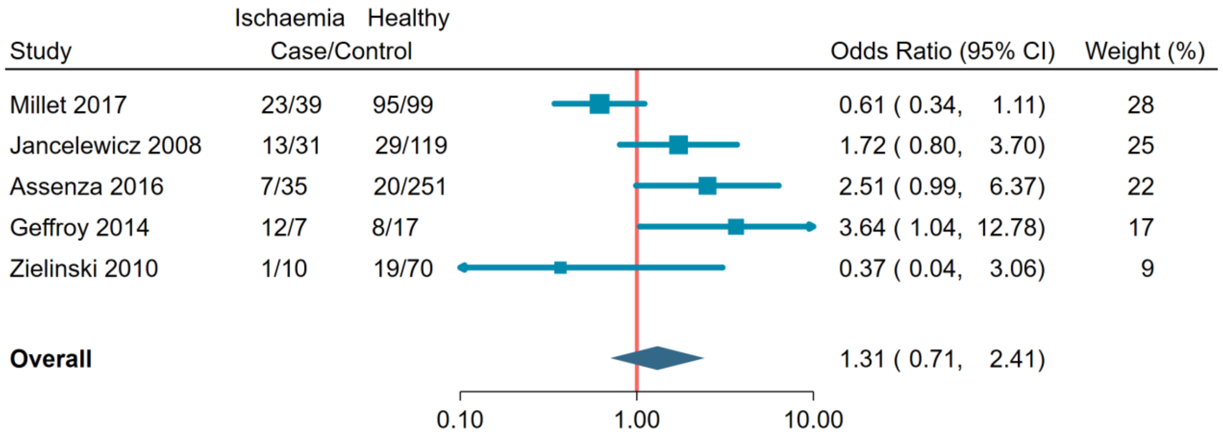

Presence of transition point:

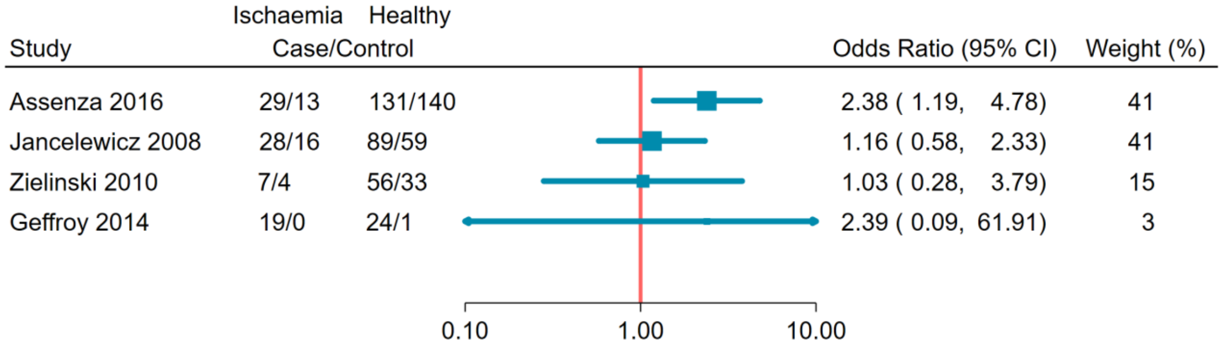

Abdominal distension:

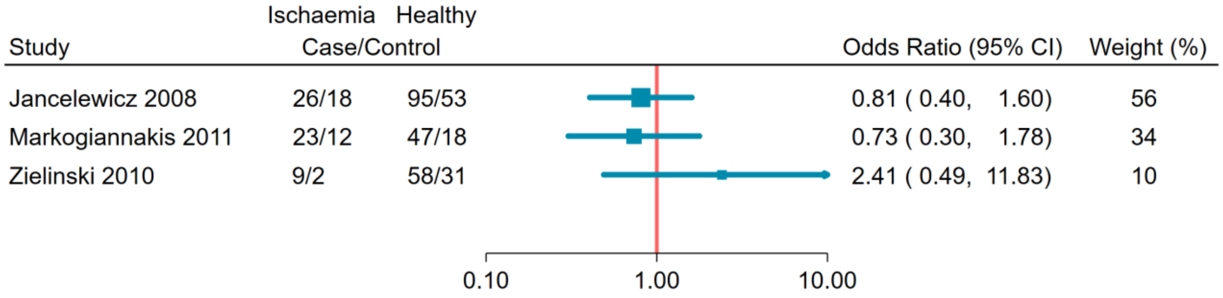

Dilated small bowel:

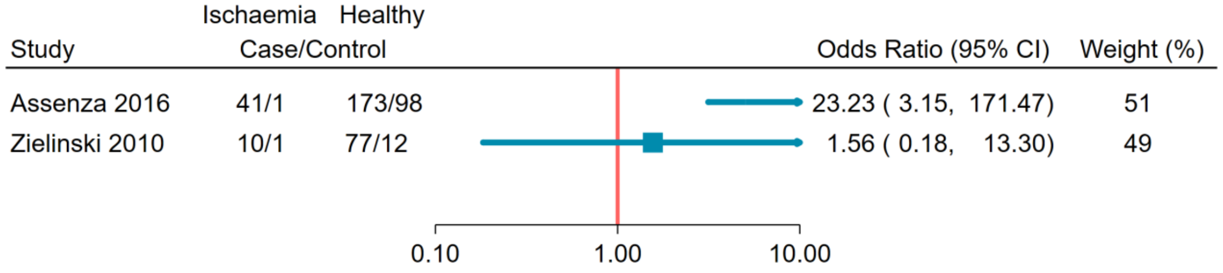

Gas-fluid levels:

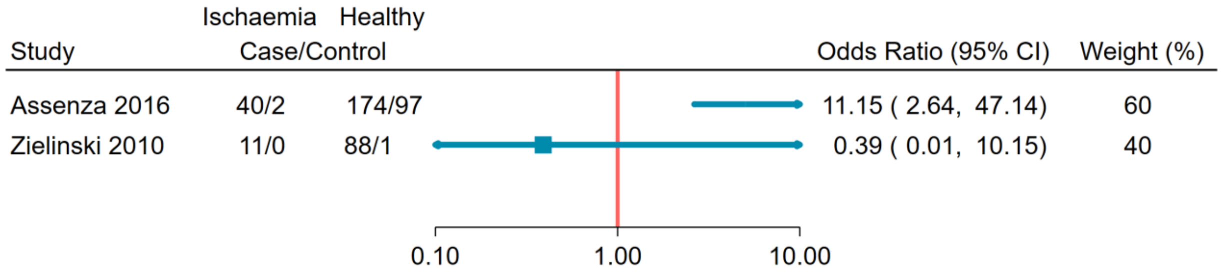

History of cardiac disease:

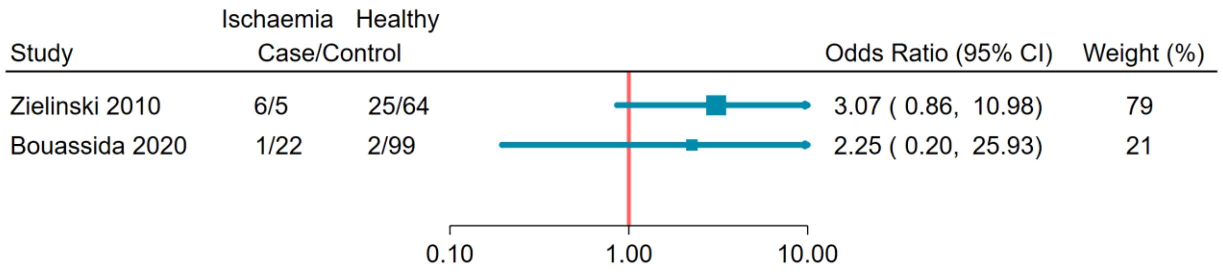

History of diabetes:

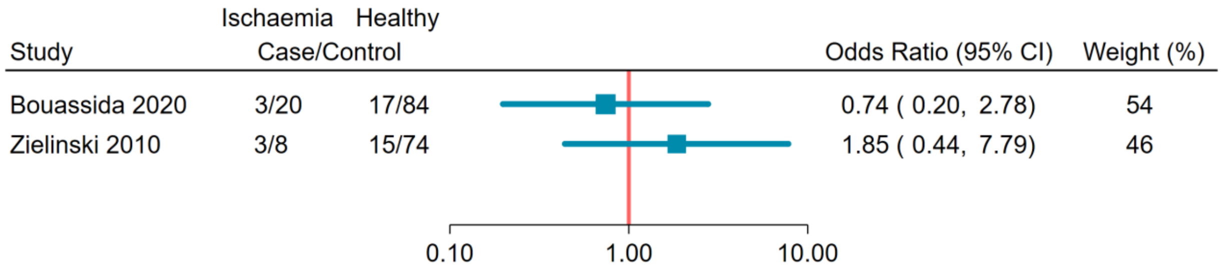

History of small bowel obstruction:

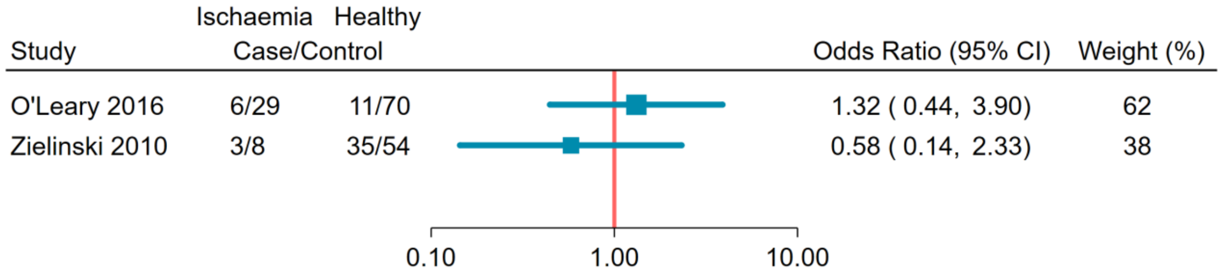

Nausea or vomiting:

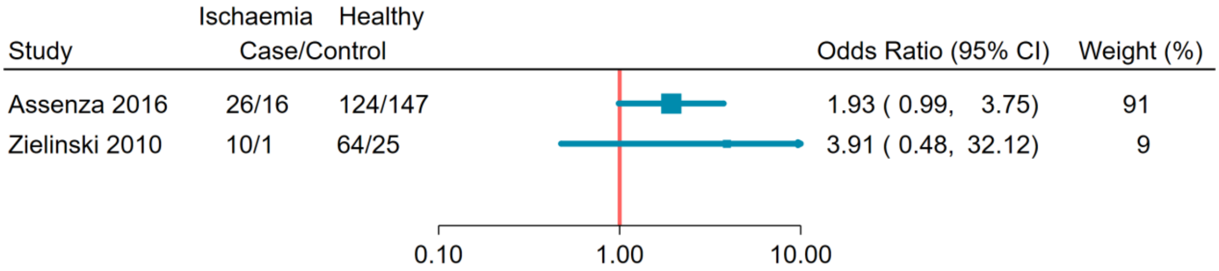

Pain:

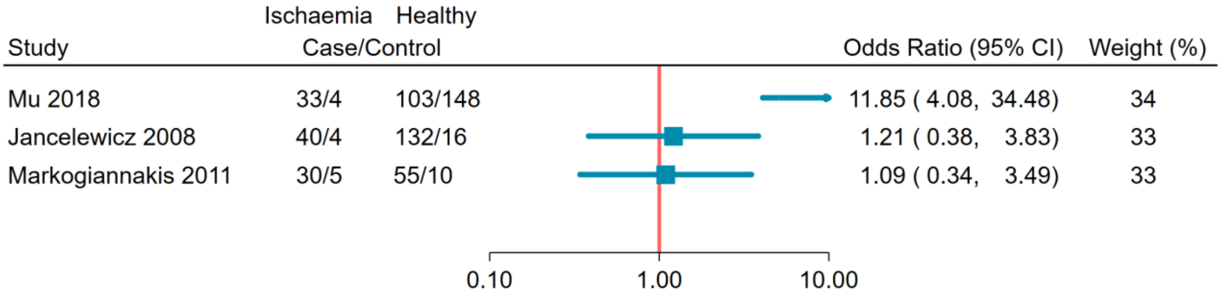

Peritonism:

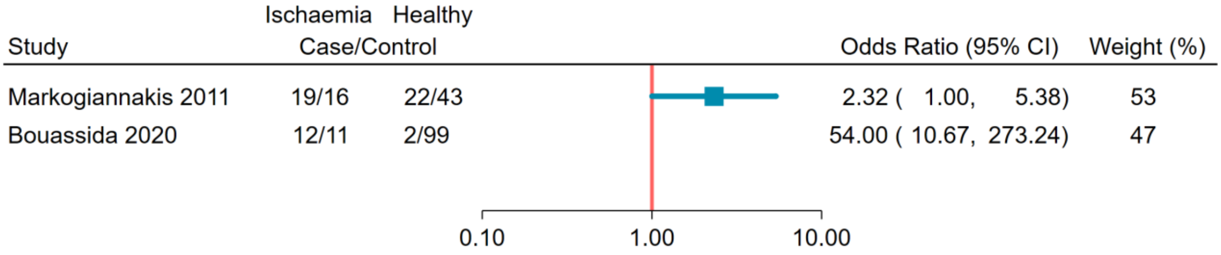

Tachycardia:

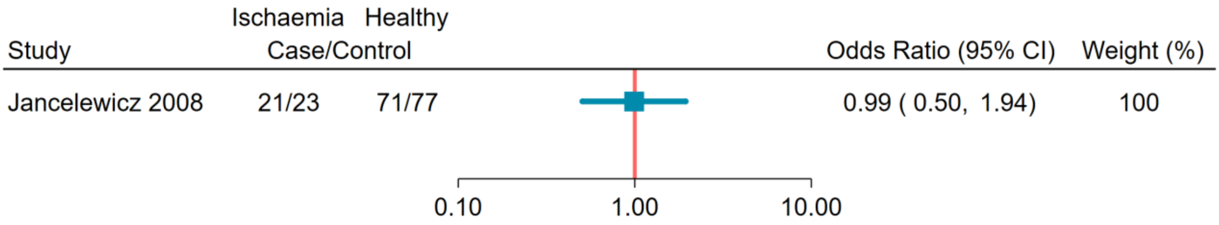

BUN:

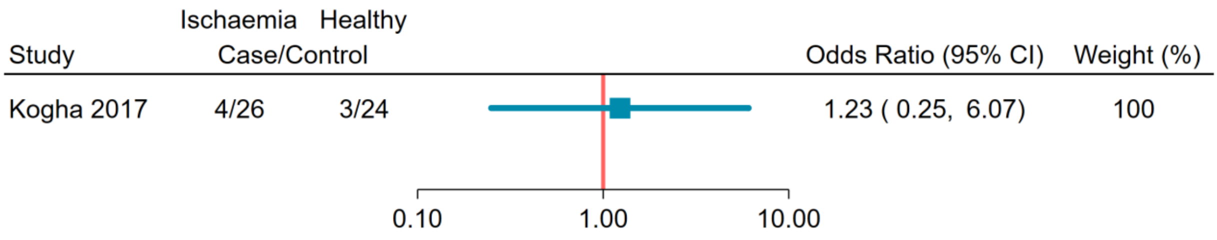

CT signs of bowel necrosis:

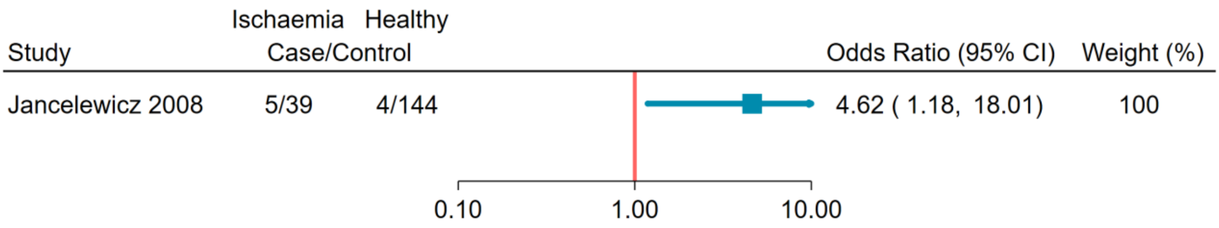

CT whirl sign:

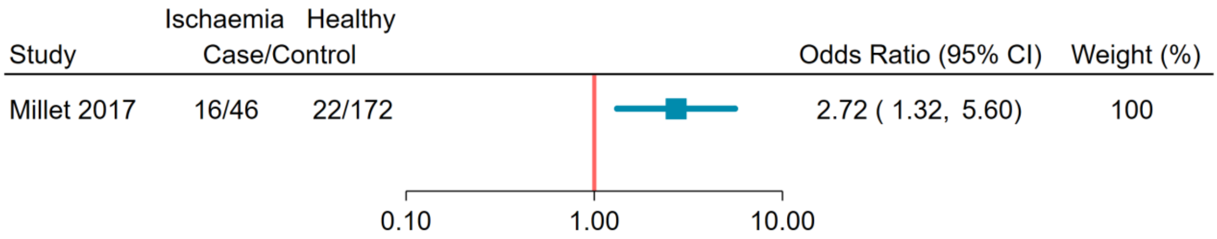

Elevated CRP:

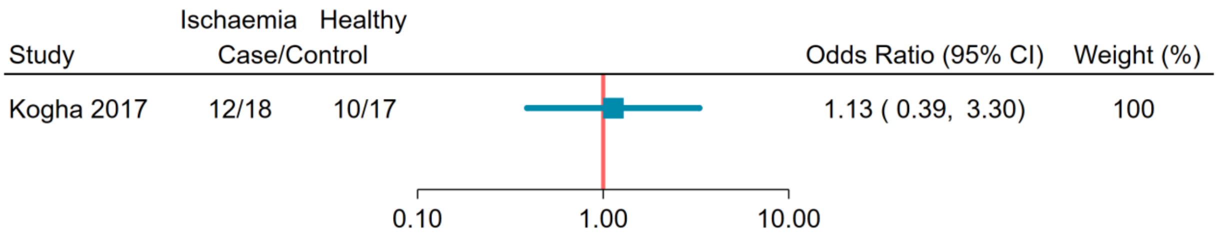

Pyrexia:

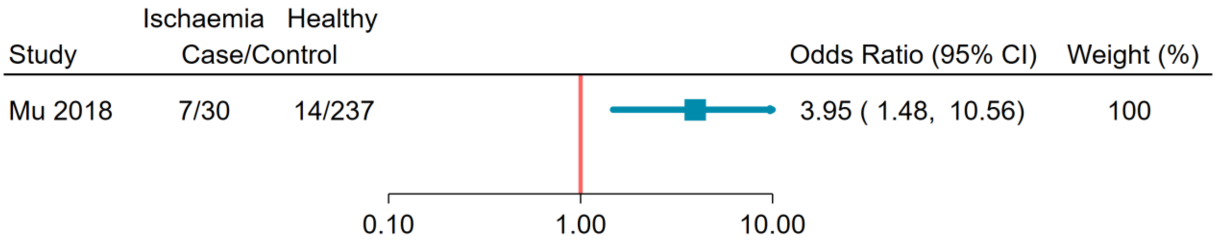

Obstipation:

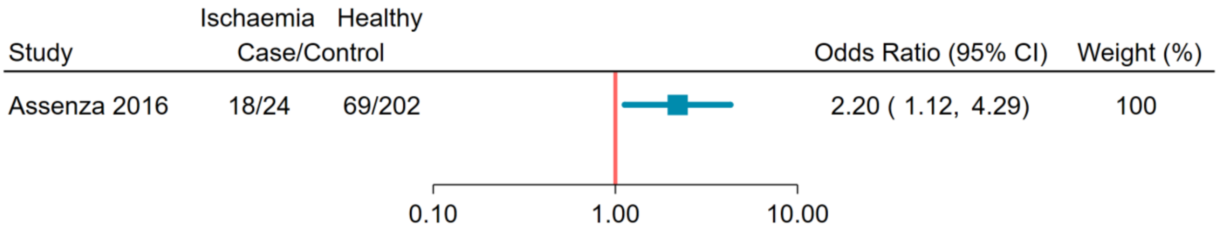

Supplement: Supplementary file 1 — Supplementary file1 (PDF 5539 KB) [file 330_2023_10421_MOESM1_ESM.pdf]
